# Rise and fall of †Pycnodontiformes: Diversity, competition and extinction of a successful fish clade

**DOI:** 10.1002/ece3.7168

**Published:** 2021-01-19

**Authors:** John J. Cawley, Giuseppe Marramà, Giorgio Carnevale, Jaime A. Villafaña, Faviel A. López‐Romero, Jürgen Kriwet

**Affiliations:** ^1^ Faculty of Earth Science, Geography and Astronomy Department of Palaeontology University of Vienna Geozentrum Vienna Austria; ^2^ Dipartimento di Scienze della Terra Università degli Studi di Torino Torino Italy; ^3^ Centro de Investigación en Recursos Naturales y Sustentabilidad Universidad Bernardo O'Higgins Santiago Chile; ^4^ Paleontological Institute and Museum University of Zurich Zurich Switzerland

**Keywords:** competition, diversity, extinction, morphospace analysis, niche partitioning, Pycnodontiformes

## Abstract

†Pycnodontiformes was a successful lineage of primarily marine fishes that broadly diversified during the Mesozoic. They possessed a wide variety of body shapes and were adapted to a broad range of food sources. Two other neopterygian clades possessing similar ecological adaptations in both body morphology (†Dapediiformes) and dentition (Ginglymodi) also occurred in Mesozoic seas. Although these groups occupied the same marine ecosystems, the role that competitive exclusion and niche partitioning played in their ability to survive alongside each other remains unknown. Using geometric morphometrics on both the lower jaw (as constraint for feeding adaptation) and body shape (as constraint for habitat adaptation), we show that while dapediiforms and ginglymodians occupy similar lower jaw morphospace, pycnodontiforms are completely separate. Separation also occurs between the clades in body shape so that competition reduction between pycnodontiforms and the other two clades would have resulted in niche partitioning. Competition within pycnodontiforms seemingly was reduced further by evolving different feeding strategies as shown by disparate jaw shapes that also indicate high levels of plasticity. Acanthomorpha was a teleostean clade that evolved later in the Mesozoic and which has been regarded as implicated in driving the pycnodontiforms to extinction. Although they share similar body shapes, no coeval acanthomorphs had similar jaw shapes or dentitions for dealing with hard prey like pycnodontiforms do and so their success being a factor in pycnodontiform extinction is unlikely. Sea surface temperature and eustatic variations also had no impact on pycnodontiform diversity patterns according to our results. Conversely, the occurrence and number of available reefs and hardgrounds as habitats through time seems to be the main factor in pycnodontiform success. Decline in such habitats during the Late Cretaceous and Palaeogene might have had deleterious consequences for pycnodontiform diversity. Acanthomorphs occupied the niches of pycnodontiforms during the terminal phase of their existence.

## Graphical abstract

Pycnodontiform lower jaws and body shape separate from ecologically similar fish clades in a morphospace analysis suggesting minimal competition between these groups. Pycnodontiform diversity appears to be little affected by abiotic factors such as sea level and climate but reef loss may be a more significant factor playing into the success of this clade. Eocene acanthomorph teleosts overlap with pycnodontiforms in body shape but have no overlap in diet so competition between these two groups unlikely factor in the decline of pycnodontiforms.

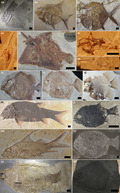

## 1 INTRODUCTION

Neopterygii (“new fins”) is a successful lineage of ray‐finned fishes consisting today of three monophyletic groups: the Ginglymodi (gars), Halecomorphi (bowfin) and Teleostei (teleosts). Ginglymodi and Halecomorphi are more closely related to each other than either is to teleosts, forming the clade Holostei (Grande, 2010). While modern holosteans are significantly depauperate (eight species; Grande, 2010) in comparison to the speciose teleosts (over 32,000; López‐Arbarello & Sferco, 2018), they exhibited large diversities of forms throughout the Mesozoic.

While stem neopterygians still are controversial (Friedman, 2015), the origin of crown Neopterygii can be traced back to the Early Triassic, and subsequent radiations occurred in the Middle to Late Triassic (Romano et al., 2014; Tintori, 1998). Several of the most successful neopterygian lineages originated in the Late Triassic such as dapediiforms (Tintori, 1983) and pycnodontiforms (Tintori, 1981). One particular factor that contributed to the radiation of neopterygians at this early stage of their evolution was their successful adaptation to different diets. The neopterygian fish fauna of the Late Triassic Zorzino Limestone in Italy, for instance, comprises dapediiforms and pycnodontiforms with short and stout jaws and massive crushing teeth likely specialized for durophagy, whereas less derived actinopterygians such as saurichthyids and birgeriids were predominantly piscivorous representing top predators in their ecosystem (Argyriou et al., 2018; Lombardo & Tintori, 2005; Tintori, 1998). Already the earliest pycnodontiforms and dapediiforms included shell‐crushing forms. Ginglymodians conversely developed similar adaptations earlier in the Mid‐Triassic and one species, †*Ticinolepis crassidens* is the earliest example of a durophagous neopterygian (López‐Arbarello et al., 2016). All three lineages originated in marine habitats but during the Mesozoic they experienced a variety of evolutionary trajectories including adaptations to new diets, habitats and even more estuarine or freshwater environments that hypothetically also controlled their success and demise.

Durophagous lineages also played an important role in the evolutionary arms escalation between shell‐crushing predators and their armored prey that characterizes the Mesozoic Marine Revolution (MMR) (Marramà et al., 2016a; Vermeij, 1977) and yet it is unknown to what degree niche partitioning played a role to take advantage of this abundant food resource and/or how severe competition occurred between fishes feeding on similar prey. According to the competition exclusion principle, complete competition between sympatric species within a Darwinian diversity‐dependence model cannot exist, resulting in the extinction of the inferior competitor, which is considered a dominant factor influencing macroevolution and diversity patterns of organisms (Hardin, 1960; Rabosky, 2013; Silvestro et al., 2015). Competition can be reduced or completely avoided by various means such as, for example, adapting to different food preferences, substrate occupation, shifts in microhabitat utilization, different daily cycles or behavioral patterns resulting from natural selection (Ebersole, 1985; Hector & Hooper, 2002; Vacher et al., 2016). Characters, which are assumed to have diverged in the past (Hector & Hooper, 2002), thus are crucial to identify niche overlap and related competition patterns but also postcompetitive (realized) ecological niche differentiation. While niche partitioning patterns are generally well documented for modern vertebrates, identifying niche partitioning patterns in extinct vertebrates is challenging (Frederickson et al., 2018), also because even extreme morphological character divergences might not prove competition in the past (Zaret & Rand, 1971). Ecological variation nevertheless generally is assumed being reflected in abundant morphological specializations. Functional traits therefore can provide a better understanding for niche partitioning and competition not only in extant, but also in extinct vertebrates (Anderson, 2008; MacLaren et al., 2016). In this context, the morphology of the lower jaw is constrained from a functional perspective providing a strong correlate for feeding function and thus for inferring diet adaptations and ecological performances (Hill et al., 2018; Neenan et al., 2014), while body shape is constrained by environmental factors representing a suitable proxy for habitat occupation (Aguilar‐Medrano, 2013; Huie et al., 2019). These proxies in combination allow identifying environmental demands of fishes and inform about possible competition patterns between fishes occupying same or at least similar habitats. This subsequently enables reconstructing evolutionary pathways of co‐occurring fishes that may result in success or failure of major clades. Analyzing competitions between organisms in deep‐time has the potential to provide important information about macroevolutionary patterns and for better understanding why some groups, such as the †Pycnodontiformes forming the focus of this study were very successful but nevertheless went extinct.

**FIGURE 1:**
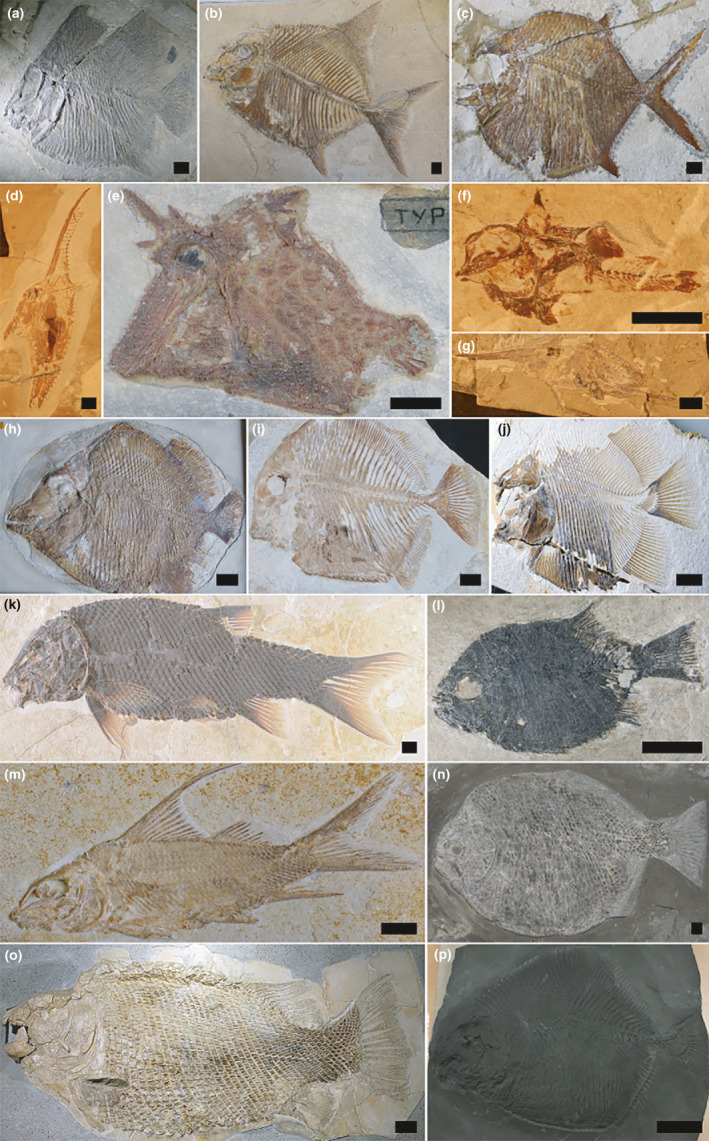
Diversity of pycnodontiform fishes, ginglymodians and dapediiforms. (a) †*Brembodus ridens* MCSNB 4901 (b) †*Proscinetes elegans* BSPG AS I 1213 (c) †*Gyrodus hexagonus* BSPG 1989 XII 110 (d) †*Gebrayelichthys uyenoi* CLC S‐538 (e) †*Ichthyoceros spinosus* MNHN HAK 106 (f) †*Corusichthys megacephalus* CLC S‐596 (g) †*Gladiopycnodus karami* CLC S‐393 (h) †*Arduafrons prominoris* NHMUK P.8658 (i) †*Akromystax tilmachiton* MNHN NRA 95 (j) †*Piranhamesodon pinnatomus* JME‐ETT4103 (k) †*Macrosemimimus fegerti* JME‐ETT 854 (l) †*Dandya ovalis* MCSNB 3463 (m) †*Propterus microstomus* BSPG 2011.I.139 (n) †*Dapedium pholidotum* SMNS 54053 (o) †*Scheenstia maximus* SMF P.2386 (p) †*Sargodon tomicus* MCSNB 10145. Scale bar for †*Arduafrons prominoris* and †*Piranhamesodon pinnatomus* is 2 cm. Scale bar for †*Scheenstia maximus* is 10 cm. Scale bar for †*Sargodon tomicus* is 5 cm. Scale bar for all other taxa is 1 cm

The overarching goal of this study is to evaluate the success but also final demise of pycnodontiform fishes, which represented the major marine actinopterygian elements from the Late Triassic to Palaeogene. To investigate possible competition relationships and resulting niche partitioning or extinction patterns indicating success or failure of taxa, we analyzed potential competition between major lineages of Mesozoic and Palaeogene nonteleostean neopterygian fishes that, due to their jaw and tooth morphology, are considered durophagous, the †Pycnodontiformes, †Dapediiformes, and Ginglymodi (Figure 1), and †Phyllodontidae representing extinct durophagous teleosts. We consequently used body and jaw morphospace and diversity analyses, respectively for evaluating the phenotypic evolution and ecological context based on competition and niche partitioning patterns in deep‐time. We also tested whether competition with acanthomorph teleosts existed because one hypothesis advocates that the rise of teleosts, especially the acanthomorphs, with their extremely successful refinements related to locomotion (Dewar & Graham, 1994) and feeding (Wainwright et al., 2012, 2015) among other adaptations (Davies & Hew, 1990; Wegner et al., 2015) might have triggered the extinction of pycnodontiforms. For this, we compared pycnodontiform and acanthomorph body plans using a geometric morphometric approach to analyze possible niche overlap between these groups in the Late Cretaceous and Palaeogene. Additionally, we also tested if abiotic factors played a role in pycnodontiform success and extinction. We correlated three abiotic factors (sea surface temperature (SST), sea level and reef area) with pycnodontiform diversity patterns to identify what role environmental changes might have played in the decline of this fish clade.

### 1.1 Major Mesozoic and Palaeogene nonteleostean neopterygian clades

†Dapediiformes was a modestly diverse neopterygian clade exhibiting a small temporal range from the Late Triassic (Norian) (Lombardo & Tintori, 2005) to their disappearance in the Late Jurassic (Tithonian) (Szabó & Pálfy, 2020). These fishes were quite conservative in body shape with deep, disk‐shaped bodies and jaws comprising stout, compact elements bearing blunt chisel‐like teeth. They are typically considered generalist durophages and this combination of opportunism and ability to access hard prey items is considered a major factor in their success after the end‐Triassic extinction (Lombardo & Tintori, 2005; Smithwick, 2015). Unlike ginglymodians, they were predominantly marine, but with some freshwater representatives (e.g., †*Hemicalypterus* with multicuspid scraping teeth; Gibson, 2015).

Ginglymodians were among the most successful Mesozoic neopterygian clades and their rates of body size and shape evolution can match and even exceed that of teleosteans throughout the Mesozoic (Clarke et al., 2016). During the Mesozoic, ginglymodians made several freshwater incursions and were exclusively restricted to freshwaters by the Late Cretaceous (Cavin, 2010). After their migration into freshwaters, new trophic adaptations appeared in ginglymodians allowing them to occupy new ecological niches such as suction feeding on small invertebrates (Thies, 1996) and herbivory/detritivory (Cavin et al., 2013). From the late Early Cretaceous onwards, a new and significant family of ginglymodians, the gars (Lepisosteidae) appeared, survived the K/Pg extinction event and are the only ginglymodians still living today. Their elongated jaws with sharp, needle‐like teeth indicate a shift to piscivory. Only a single lepisosteid, the middle Eocene †*Masillosteus,* most likely was durophagous based on its shortened jaws and large, blunt teeth (Micklich & Klappert, 2001). Whether this is an “intermediate” form between more typical durophagous ginglymodians and modern gars or if †*Masillosteus* had secondarily developed durophagy is still ambiguous. A phylogenetic analysis of lepisoteids (Cavin, 2010) identified †*Massilosteus* as sister of piscivorous forms such as †*Obaichthys*, †*Oniichthys* and *Lepisosteus*.

†Pycnodontiformes included laterally compressed, deep‐bodied fishes that were mostly confined to near‐coastal, often structured marine habitats. Their fossil record spans 175 million years from the Late Triassic (Norian) to the late Eocene (Priabonian) (Voss et al., 2019), but their early fossil record is patchy with three genera from the Late Triassic, which are completely preserved, while only isolated teeth and jaws are present in the Early to Mid‐Jurassic (Stumpf et al., 2017) with very rare exceptions of better preserved yet incomplete specimens (Ebert & Kölbl‐Ebert, 2018). Late Jurassic records range from isolated teeth to holomorphic individuals and by now, their taxonomic diversity seemingly has increased substantially (Agassiz, 1833, 1834; Ebert, 2016, 2020; Ebert et al., 2017; Frickhinger, 1991; Gistl, 1848; Kölbl‐Ebert et al., 2018; Wagner, 1862). This continues to increase during the Cretaceous with its peak in both species richness and morphological disparity occurring in the Cenomanian (Marramà et al., 2016a). Irrefutably, †Pycnodontiformes is the only durophagous nonteleostean lineage present in Late Cretaceous seas due to the Late Jurassic extinction of dapediiforms and the complete adaptation to freshwaters by ginglymodians in the early Late Cretaceous (Cavin, 2010). The impact of the K/Pg extinction event was severe for pycnodontiforms and diversity was afterwards far smaller than before and they never attained diversity patterns as before the K/Pg boundary until they finally disappeared in the late Eocene.

Pycnodontiforms predominantly had powerful jaws with well‐developed coronoid processes for adductor muscle attachment and rows of molariform crushing teeth (Kriwet, 2001, 2005). The premaxillae and dentalosplenials typically had more gracile, incisiform teeth for removing prey items from the substrate transferring it to the crushing molariform teeth. This is an ecomorphological system that has clearly been successful for pycnodontiforms given their long presence in the fossil record. Moreover, contrary to ginglymodians, they seemingly had conserved their ancestral jaw morphologies until their extinction while different feeding modes evolved throughout their evolutionary history (Kölbl‐Ebert et al., 2018; Marramà et al., 2016a; Taverne & Capasso, 2013a; Vullo et al., 2017, 2019).

### 1.2 Mesozoic and Palaeogene durophagous teleostean clades

Durophagous teleosts potentially represent competitors for durophagous nonteleostean clades, especially pycnodontiforms, for prey. Durophagous teleosts, however, are quite rare in pre‐Cenozoic times. Extinct durophagous albuliforms †Phyllodontidae are characterized by stacked (phyllodont) oral toothplates with smooth, rounded teeth (Estes & Hiatt, 1978) and ranged from the Late Cretaceous (Campanian) to the end of the late Eocene (Priabonian) (Vullo et al., 2009; Westgate, 2001). They thus might have competed with pycnodontiforms over similar prey resources. However, their fossil record is extremely fragmentary with only five known genera and ca. 17 species that are based on teeth and preserved tooth plates (e.g., Estes, 1969; Halliday et al., 2016), thus making them unsuitable for morphospace analyses.

Within acanthomorph fishes, the fossil evidence of durophagous taxa is mostly restricted to several lineages pertaining to the speciose percomorph clade, which used oral and/or pharyngeal jaws (e.g., Grubich, 2003) to process hard prey. Although some isolated teeth would suggest an early Palaeocene existence of durophagous percomorphs (see, e.g., Arambourg, 1952), articulated skeletal remains (e.g., gymnodont tetraodontiforms, labrids, sparids) or isolated beak‐like jaws (e.g., gymnodont tetraodontiforms, oplegnathids) of durophagous taxa appeared only during the early Eocene (e.g., Bannikov & Carnevale, 2010, 2012; Bannikov et al., 2017; Carnevale, 2015; Cione et al., 1994; Santini et al., 2014; Tyler, 1980).

## 2 MATERIALS AND METHODS

### 2.1 Systematic groups and specimens analyzed

This study focuses on four major neopterygian clades: †Pycnodontiformes, †Dapediidae, Ginglymodi, which were prominent components of bony fish faunas during the Mesozoic, and Acanthomorpha, which were the dominant bony fish clade during the Cenozoic. We also evaluated the impact of extinct phyllodontids on pycnodontiform diversity patterns to identify if possible ecological competition occurred between both groups.

Pycnodontiforms represent a well‐defined monophyletic group (see Poyato‐Ariza & Wenz, 2002 and Ebert & Kölbl‐Ebert, 2018), but the intrarelationships of various taxa and groups remain debated. Nevertheless, †Pycnodontiformes includes several monophyletic groups at family level such as †Brembodontidae, †Coccodontidae, †Gebrayelichthyidae, †Gladiopycnodontidae, †Gyrodontidae, †Mesturidae and †Pycnodontidae, for which complete lower jaws or holomorphic specimens are preserved. The exact systematic position of †*Piranhamesodon pinnatomus* is still unresolved, despite it being supposedly a basal member of †Pycnodontoidei (Kölbl‐Ebert et al., 2018). We therefore consider †*P. pinnatomus* as “family incertae sedis” along with *Apomesodon*, *Macromesodon* and “*Eomesodon*”. Other possible “piranha‐like” pycnodontiforms such as the †Serrasalmimidae (Vullo et al., 2017) are excluded here, as only very fragmentary jaws are known, which do not allow employing geometric morphometric approaches.

†Dapediidae was originally assigned to the order †Semionotiformes (Lehman, 1966; Thies & Hauff, 2011) but recently identified to represent a distinct order, †Dapediiformes (López‐Arbarello, 2012; Thies & Waschkewitz, 2016). This order represents either the sister of ginglymodians (Gibson, 2016) or Holostei (López‐Arbarello & Sferco, 2018). Regardless, †Dapediiformes is considered here as a separate group from either †Pycnodontiformes or Ginglymodi. Ginglymodians included in our study members of the families, †Callipurbeckidae, †Macrosemiidae and †Lepidotidae.

Acanthomorphs (spiny‐rayed teleosts) were included in the full‐body shape analysis to identify any possible competition patterns with pycnodontiforms in habitat occupancy as expressed by morphospace occupation. Since acanthomorphs only truly started to diversify at the end of the Cretaceous, representatives from this period and the Palaeogene are included here. Additionally, their absence from the lower jaw analysis is due to the recurrent use of pharyngeal jaws by acanthomorph groups for eating hard prey (see Grubich, 2003), while some lineages such as sparids apparently evolved considerable oral jaw adaptations for durophagy only during the Oligocene (Santini et al., 2014) after pycnodontiforms went extinct. Thus, it is impossible that they were competing with pycnodontiforms in terms of prey acquisition conversely to ginglymodians and dapediiforms.

We used two time bins to evaluate the ecological relationships between pycnodontiforms and acanthomorphs represented by the early Late Cretaceous fossil lagerstätten of Haqel, Lebanon and the Eocene Fossil Lagerstätte of Monte Bolca, Italy, as in these localities pycnodontiforms had their taxonomic diversity peak (Haqel) or includes one of their final occurrences in the fossil record (Monte Bolca). These two time bins are important because they can inform about morphospace overlap between both suggesting competition for habitat, but also if acanthomorphs contributed to pycnodontiform extinction. Data for acanthomorphs from Bolca are based on Marramà et al. (2016b), Marramà et al. (2016c), while data for those from Haqel were obtained from Gayet et al. (2012).

A total of 67 species (one specimen per species) with 40 belonging to †Pycnodontiformes, seven to †Dapediiformes and 20 to Ginglymodi allowed to capture the functional diversity of the lower jaws in articulated specimens. For the full‐body morphospace analysis, a total of 274 taxa (one specimen per species) were suitable, because they displayed all necessary landmark positions: 60 species belonging to †Pycnodontiformes, 10 to †Dapediiformes, 19 species to Ginglymodi and 185 to Acanthomorpha.

Specimens housed in the following museum collections were used: **AMNH,** American Museum of Natural History, New York, USA; **BSPG,** Bayerische Staatssammlung für Paläontologie und Geologie, Munich, Germany; **CLC**, Luigi Capasso collection, Chieti, Italy; **JME**, Jura‐Museum Eichstätt, Germany; **MCSNB**, Museo Civico di Storia Naturale “E. Caffi”, Bergamo, Italy; **MCSNV,** Museo Civico di Storia Naturale di Verona, Verona, Italy; **MHNL,** Musée des Confluences, Lyon, France; **MNHN**, Muséum National d’Histoire Naturelle, Paris, France; **MPUM**, Museo Paleontologico dell’Università degli Studi di Milano, Milan, Italy; **NHMUK,** Natural History Museum, London, UK; **NHMW,** Naturhistorisches Museum Wien, Vienna, Austria; **NRM,** Naturhistoriska Riksmuseet, Stockholm, Sweden**; SMF,** Senckenberg Forschungsinstitut und Naturmuseum, Frankfurt, Germany; **VFKO,** Verein der Freunde und Förderer des Naturkundemuseums Ostbayern, Germany.

All taxa used for analysis are marine, as this gives a more accurate understanding of how these clades functioned in a singular ecosystem. Freshwater taxa were excluded because most freshwater pycnodonts (with the exception of rare occurrences such as, e.g., the Early Cretaceous Las Hoyas pycnodonts) are represented by isolated dental remains only, and contemporaneous freshwater ginglymodians were piscivores. The probably durophagous lepisosteid, †*Masillosteus* from the Eocene certainly never competed with pycnodonts for resources since pycnodonts never entered the Messel lake.

### 2.2 Geometric Morphometrics

**FIGURE 2:**
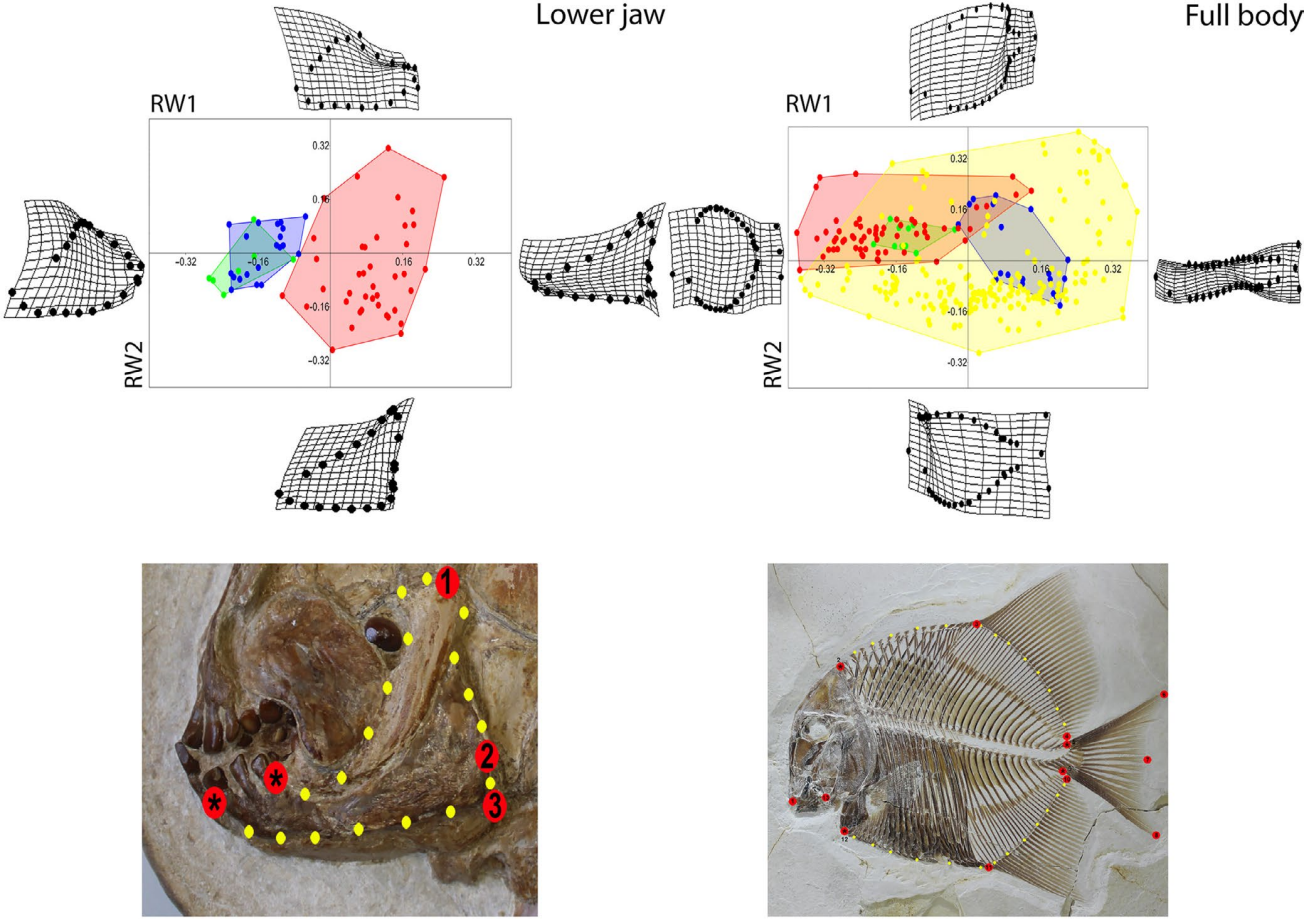
Morphospace of neopterygian fish groups based on landmark analysis. Deformation grids illustrate the shapes lying at extreme values along each axis. Morphospaces of each group is color coded: Green—†Dapediiformes, Blue—Ginglymodi, Red—†Pycnodontiformes, Yellow—Acanthomorpha. Lower jaw morphospace based on the first two RW axes together accounting for 55.39% of the overall shape variation (RW1: 31.34%, RW2: 24.05%). Lower jaw landmarks and semi‐landmarks as shown on †*Neoproscinetes penalvai* (BSPG 1999 I 30) from the Early Cretaceous of the Santana Formation, Brazil, for the geometric morphometric analysis. Full‐body morphospace based on the first two RW axes together accounting for 65.47% of the overall shape variation (RW1: 42.05%, RW2: 23.41%). Landmarks and semi‐landmarks used on the full body (The pycnodontiform †*Turbomesodon relegans* (JME‐ETT119) from the Upper Jurassic of Ettling, Germany, pictured here) for the geometric morphometric analysis. Landmarks are numbered and are in red and semi‐landmarks are in yellow. Landmarks with an asterisk are anchor points for the intervening semi‐landmarks

For the lower jaw analysis, three landmarks were chosen corresponding to functional points (Figure 2), which correlate with the linear measurements Bellwood (2003) used to construct closing and opening lever ratios to determine the biomechanics of jaw movements. Two additional landmarks at the anterior and posterior tips of the dentary tooth row defining the biting/chewing area represent anchor points for 18 semi‐landmarks that capture the overall shape of the jaw. Landmarks coded for the lower jaw are as follows: (a) the highest point of the lower jaw where the adductor mandibulae muscles (particularly the A2 muscle) insert, which determines bite force; (b) the articulation point where the articular of the lower jaw abuts the quadrate and is the fulcrum of the lower jaw around which the jaw closes during feeding (Westneat, 1994, 2003); (c) the most postero‐ventral margin of the jaw where the interopercular‐mandibular ligament attaches that mediates rotation of the lower jaw about the quadrato‐mandibular joint by caudal motion resulting in depression of the lower jaw. In the case of pycnodontiforms, a muscle mass from the paired prearticulars expands to the ceratohyal and epihyal (Kriwet, 2001a), which is functionally similar to the interopercular‐mandibular ligament in attachment point. Landmarks and semi‐landmarks were digitized on photos of lower jaws using the software TPSdig (Rohlf, 2005). Generalized Procrustes Analysis (GPA) was applied using the software TPSRelw (Rohlf, 2003) to the landmark coordinates for removing effects of different configuration such as size, location and orientation (Rohlf & Slice, 1990; Zelditch et al., 2012). Semi‐landmarks were treated to slide treatment to reduce the bending energy of the curves (Gunz & Mitteroecker, 2013). The Principal Component Analysis (PCA) was performed on the new Procrustes coordinates through TPSRelw, which also reveals how jaw shape changes along the axes through deformation grids.

For the full‐body morphospace analysis, a total of 13 landmarks and 26 semi‐landmarks were digitized (Figure 2), which are the same used by Marramà et al. (2016b). As well as comparing shape between each clade in an overall morphospace, taxa were split into five time bins (Late Triassic, Jurassic, Early Cretaceous, Late Cretaceous and Palaeogene) to evaluate shape changes through time. Reasoning for using larger time bins rather than one‐million‐year time bins as often done is that complete pycnodont specimens displaying all landmark positions for both the lower jaw and body shape are rather rare being related to specific preservational conditions and that the exact stratigraphic age of most of these specimens is unknown or ambiguous.

### 2.3 Statistical analyses

An Analysis of Similarities (ANOSIM) (Clarke, 1993) was performed using all of the Relative Warp (RW) score axes that make up the morphological variance of the body and lower jaw shape, to establish the degree of morphological overlap between †Pycnodontiformes, Ginglymodi, †Dapediiformes and Acanthomorpha. Tests were conducted between Mesozoic families in general and each major group (†Pycnodontiformes, Ginglymodi, †Dapediiformes and Acanthomorpha) through time. The association of shape variation related to their taxonomic group was estimated with a Procrustes ANOVA using the procD.lm function from the R package geomorph (version 3.3.1). With the shape as a response and the categories (Orders, pycnodontiform families and orders through time), followed by a post hoc pairwise comparison between the least squares means with the pairwise function of the package RRPP (version 0.6.1) (Collyer & Adams, 2018).

The disparity for each group with the body and jaw landmark configuration was then compared with the pairwise function in RRPP to estimate the distance between variances, (Zelditch et al., 2012). ANOSIM could not be performed using geomorph so had to be performed using PAST 2.17c (Hammer et al., 2001).

### 2.4 Calculating pycnodontiform diversities

To further investigate potential effects of competition that other clades might have exerted on pycnodontiforms, the diversity patterns of the three major Mesozoic durophagous nonteleostean groups, †Dapediiformes (9 genera), Ginglymodi (38 genera), as well as that of teleostean †Phyllodontidae (5 genera) were calculated. The fossil occurrence dataset contains 81 pycnodontiform genera that covers the span of their fossil record (about 180 million years; Late Triassic to Late Eocene) to correlate pycnodontiform with diversity patterns of the other clades but also with abiotic factors. An exhaustive overview of the literature was performed including numerous taxonomic revisions (e.g., Ebert, 2020; Ebert et al., 2017; Koerber, 2012; Poyato‐Ariza, 2013; Poyato‐Ariza & Wenz, 2002, 2004; Taverne & Capasso, 2012, 2014a; Taverne et al., 2015, 2019; Vullo & Courville, 2014). Both body fossil and more fragmentary material (jaws, isolated dentition) occurrences were used to produce a large and robust dataset. †*Coelodus saturnus* Heckel, 1854 is used as the only representative of the genus, since the taxonomic position of all the other †*Coelodus* species has to be considered dubious. Genera were used here since preservation of fossil material often is too incomplete for unambiguous species identification and higher taxonomic units can compensate for small‐scale fluctuations in sampling by interpolating the temporal range of a taxon in the fossil record between its first and last occurrence (Smith, 2001).

Using the occurrence dataset, diversity dynamics of pycnodontiform fishes and the other clades were calculated using the R package divDyn (Kocsis et al., 2019). Genus richness, extinction and origination rates were measured in divDyn in time bins lasting one million years. Singleton occurrences were removed to reduce biases caused by the Lagerstätten effect (Lu et al., 2006), which should produce a more accurate picture of pycnodontiform diversity.

Comparing the diversity patterns of the four durophagous fish groups potentially elucidate if the success/decline of one group has a particular effect on another. In the case of †Phyllodontidae, it is an alternative way to investigate if these fish could have been potential competitors for pycnodontiforms since their poor fossil material makes them unsuitable for morphospace analysis. Stratigraphic ages for these fish groups were compiled using a combination of literature research and the Paleobiology Database (PaleoDB; http://paleobiodb.org).

Pycnodontiforms were predominantly durophagous and we therefore also included the diversity patterns of potential prey items to examine potential correlations with patterns of pycnodonts. For this, we established diversity patterns and origination and extinction rates of shelled invertebrate taxa from the Palaeogene (Palaeocene‐Eocene): molluscs (2,068 genera), echinoderms (221 genera), bryozoans (276 genera) and brachiopods (88 genera). All cephalopods lacking a shell (Neocoleoidea) were excluded from this analysis, as these were unlikely to be typical prey. All data pertaining to the invertebrate groups used in this study were obtained from the Paleobiology Database.

### 2.5 Correlating pycnodontiform diversity with palaeotemperature and sea level

Pycnodontiform diversity patterns also were compared to the SST curves through time and sequence stratigraphic sea level estimates to identify possible environmental factors that might have influenced pycnodontiform diversity and their final disappearance. A particular problem with SST data is that sampling across time bins can be inconsistent and thus impact the means of particular intervals (Kelley et al., 2014). To circumvent this issue, Jouve et al. (2017) constructed a polynomial curve and the theoretical values obtained therein are used to reconstruct the temperature curve, which we will be using here. Additional SST curves (smoothing spline, two curves with three‐point moving average and weighted three‐point moving average; Jouve et al., 2017) were also used. While the data used by Jouve et al. (2017) covered the Hettangian to Rupelian, we considered measurements from the Hettangian to Priabonian only. Although this excludes the earliest records of pycnodontiform evolutionary history (Norian‐Rhaetian), it covers the vast majority of their evolutionary history. SST data was calculated by Martin et al. (2014) through the use of oxygen isotopes obtained from fish tooth enamel. These were collected from European, Middle East, American and North African localities, which would have corresponded to the Western Tethys. Fish tooth enamel is considered the best biomineral for estimating Pre‐Cenozoic marine temperatures (Lécuyer et al., 2003; Picard et al., 1998) due to its strong resistance to diagenesis (Kolodny et al., 1996; Sharp et al., 2000) and a consistent oxygen isotope composition of phosphate, which persists during long geological time scales (Kolodny et al., 1983; Lécuyer et al., 1999) due to the large and densely packed apatite crystals that comprise tooth enamel. Sea level estimates were taken from Haq et al. (1987).

## 3 RESULTS

### 3.1 Lower jaw morphospace occupation

The overall functional morphospace (combining all time bins) shows a substantial separation between †Pycnodontiformes, and the other two Mesozoic nonteleostean clades (†Dapediiformes and Ginglymodi) along the first RW axis (Figure 2). Both ginglymodians and dapediiforms, conversely, have substantial degrees of overlap as their jaws share similar morphologies and are restricted to the negative end of RW1. Negative RW1 is related to taxa with a large biting surface area, forward facing coronoid process, quadrate articulation in a concave notch and reduced jaw depth toward the posterior end. The pycnodontiforms on the positive end of RW1 reveal that their jaw morphology shows a smaller biting area, posterior position of a high coronoid process, medial articulation of the quadrate along the posterior edge of the jaw and an increased jaw depth toward the posterior end. Specimens with negative RW2 values have short and deep jaws, narrow and high coronoid processes, a medial quadrate articulation and absence of an obvious posterior process (Figure 2). Fishes on the positive end of RW2, conversely, show elongated jaws with a broad, low coronoid process, high articulation point with the quadrate and a concave notch located anteriorly to the posterior process.

†Pycnodontiformes occupy a vastly larger morphospace than either Ginglymodi or †Dapediiformes (Figure 2).

**FIGURE 3:**
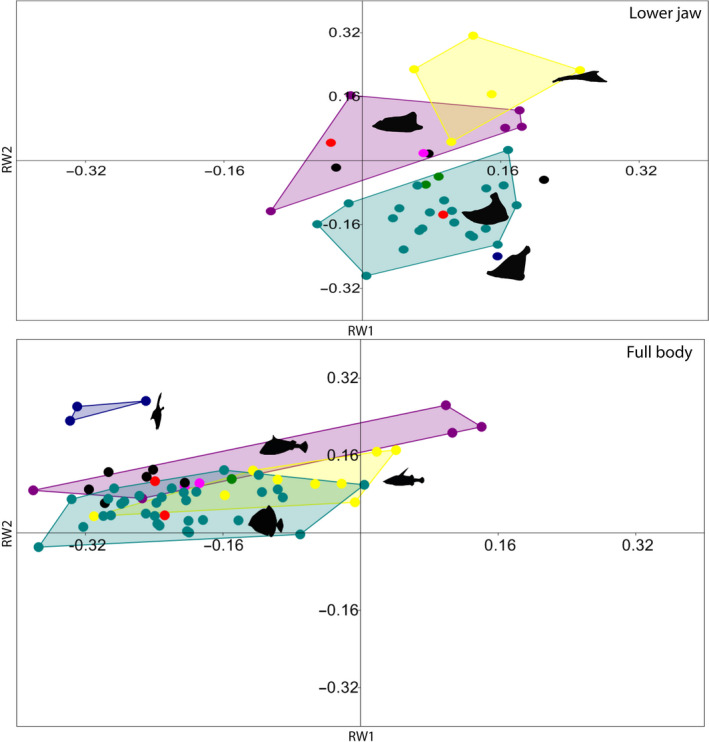
Morphospace including pycnodontiform families based on landmark analysis. Morphospace of each family is color coded: Turquoise—†Pycnodontidae, Dark blue—†Gebrayelichthyidae, , Green—†Mesturidae, Pink—†Gyrodontidae, Red—†Brembodontidae, Purple—†Coccodontidae, Yellow—†Gladiopycnodontidae, Black—“Family incertae sedis”. Silhouettes are representative of each group: †Coccodontidae—†*Coccodus armatus*, *†*Gebrayelichthyidae—†*Gebrayelichthys uyenoi*, †Gladiopycnodontidae—†*Joinvillichthys lindstroemi*, †Pycnodontidae (lower jaw)*—*†*Tepexichthys aranguthyorum*, †Pycnodontidae (full body)—†*Akromystax tilmachiton*. All full‐body pycnodontiform silhouettes are modified from Marramá et al. 2016a

**FIGURE 4:**
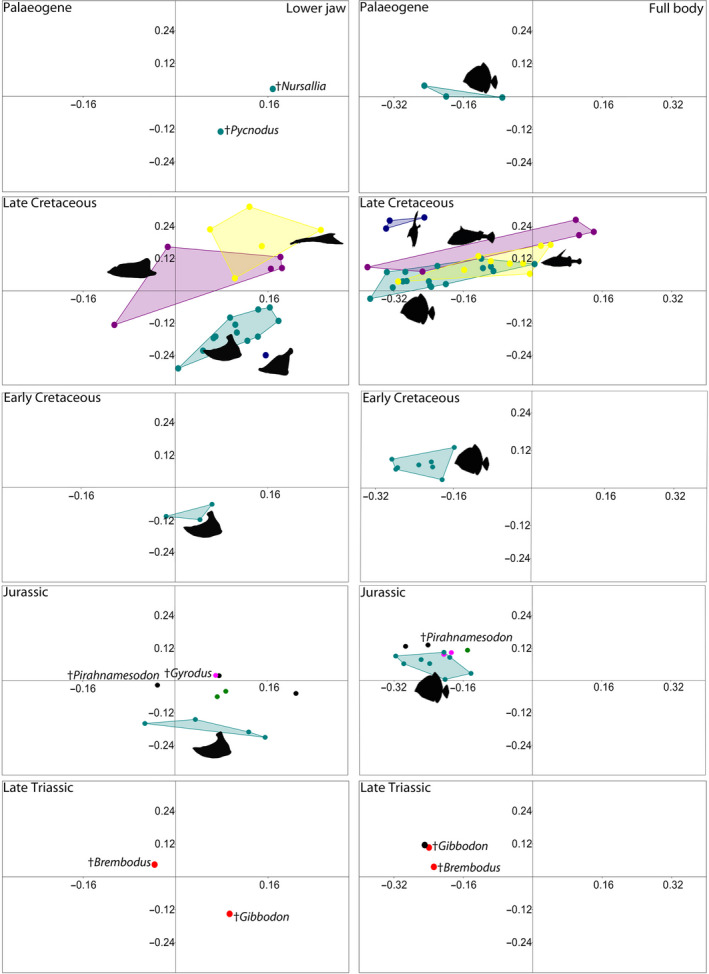
Morphospace of pycnodontiform families based on landmark analysis through five time bins. Morphospace colors and silhouettes are the same as in Figure 3

We analyzed the morphospaces of the families separately to visualize the range of disparity present within †Pycnodontiformes (Figure 3). †Pycnodontidae is mostly situated in the lower right quadrant where jaws are deep with a high coronoid processes. On the upper right quadrant are the families †Coccodontidae (with the exception of †*Trewavasia carinata* with its large dentalosplenial and high coronoid process) and †Gladiopycnodontidae, which have narrower and more elongate jaws showcasing the extremes of that jaw morphology. Further splitting of pycnodontiform families into different time bins (Figure 4) reveals that †*Piranhamesodon pinnatomus* is separated from all other taxa having a larger biting area and a lower coronoid process than pycnodontids. Gyrodontids further separate from members of other families in having the least developed coronoid process of any pycnodontiform in the Late Jurassic. In the Late Cretaceous, †Coccodontidae has no overlap with †Pycnodontidae, while †Gladiopycnodontidae has minimal overlap with coccodontids as exemplified by †*Gladiopycnodus karami*.

### 3.2 Full‐body morphospace occupation

When all four clades of neopterygians are assessed for body shape (Figure 2) we can observe that: (a) Pycnodontiforms completely overlap with dapediiforms but only minimally with ginglymodians and (b) acanthomorphs occupy all four quadrants as already found by Marramà et al. (2016b), but do not occupy the furthest left of the upper left quadrant, which is occupied by pycnodontiforms (†Gebrayelichthyidae, in particular) (Figure 2). Deep‐bodied forms occupy negative RW1 while more elongate forms occupy positive RW1. †Pycnodontiformes and †Dapediiformes are on the negative RW1 axis while ginglymodians are on the positive axis. Negative RW2 represents forms with a long anterior–posterior dorsal fin with many pterygiophores, which is anterior to the orbit and a forked caudal fin while positive RW2 includes forms with large heads and small median fins concentrated around the posterior part of the trunk and a convex caudal fin (Figure 2). The acanthomorph clades Pleuronectiformes and Tetraodontiformes represent the negative and positive ends of RW2 axis, respectively.

### 3.3 Patterns of morphospace occupation

**FIGURE 5:**
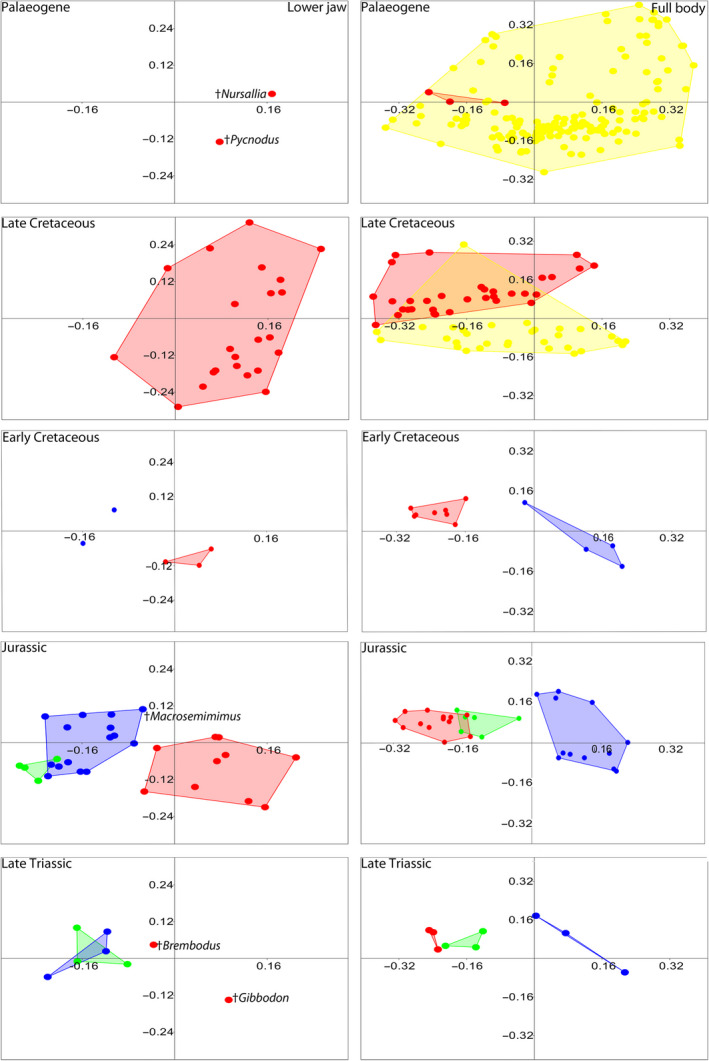
Morphospace of all four neopterygian fish groups based on landmark analysis through five time bins. Morphospace colors are the same as in Figure 2

The ANOSIM regarding the lower jaw across all three nonteleostean groups from the Mesozoic studied here clearly separates †Pycnodontiformes from †Dapediiformes and Ginglymodi while the latter two groups are barely indistinguishable. When all families are tested with ANOSIM, it shows them to be overlapped but with clear differences separating them. Finally, the ANOSIM results between each of the three major nonteleostean groups confirm the significant decrease in group overlap through time, which is evident between ginglymodians and pycnodontiforms in the Early Cretaceous (Figure 5).

In the full‐body analysis, there is a clear difference between †Pycnodontiformes and Ginglymodi, while †Dapediiformes is separated from Ginglymodi (Figure 2). The groups are significantly separated in each subsequent time bin from Late Triassic to Early Cretaceous, whereas there is more overlap from the Late Cretaceous to the Palaeogene. Of all the pycnodontiform families, †Coccodontidae has the highest levels of overlap with other families (Figure 3). This is due to the presence of †*Trewavasia* and †*Hensodon*, which have deep, rounded bodies in contrast to the typical fusiform morphology of more typical coccodontids. The finding that †*Hensodon* and †*Trewavasia* are closer to each other in the morphospace than either is to the other coccodontids either supports that both form a monophyletic group, the †Trewavasiidae of Nursall (1996) or the similarities could be due to convergent evolution in adapting to structured environments such as reefs. However, since no further phylogenetic work has been conducted up to now we follow the current hypothesis and consider these two taxa to belong to †Coccodontidae.

Procrustes ANOVA suggests group centroid separation (*p* < 0.05) between Ginglymodi and †Pycnodontiformes in regards to the lower jaw. When taxa are combined into families there is significant group centroid separation between many families. However, pairwise distances (see Data Archiving Statement for access to all data) reveal that all significant separation between families involves a family belonging to †Pycnodontiformes. Interestingly, significant group centroid separation for all three nonteleostean groups in the Mesozoic is only present in the Jurassic.

Results of group centroid separation between taxa in the context of body shape show clear patterns. Separation between all four neopterygian groups analyzed here is more significant than that seen in the lower jaw. Procrustes ANOVA shows that Mesozoic neopterygian families are well separated with †Gebrayelichthyidae and †Pycnodontidae especially being highly separate from all other families and the majority of families sampled here, respectively. Nonsignificant separation is recovered for similarly shaped pycnodontiforms such as †Brembodontidae and †Mesturidae.

Even deep‐bodied acanthomorphs such as members of †Pycnosteroididae are significantly separated from pycnodontids with them positioned along the negative end of the RW2 axis and †Pycnodontidae being found along the positive end. Acanthomorph families represented by single taxa (e.g., †Pletocretacicidae) are significantly separated from pycnodontids and other families. Considering all four groups through time, group centroid separation increases continuously but starts to decrease again in the Palaeogene when both pycnodontiform diversity and disparity patterns are dwarfed by acanthomorphs.

### 3.4 Morphological disparity

†Pycnodontiformes displays the highest morphological disparity of the lower jaw among the three nonteleostean groups tested followed by Ginglymodi and then †Dapediiformes. When considering the morphological disparity within pycnodontiform families, it is evident that coccodontids have the most disparate jaws while pycnodontids are the least disparate. Species richness and morphological disparity do not correlate in these results with speciose †Pycnodontidae (23 species) being the least disparate group while species‐poor †Coccodontidae (5 species) have the highest disparity due to the placement of the “trewavasiids” (†*Trewavasia* and †*Hensodon*) within †Coccodontidae.

Morphological disparity of the lower jaws of pycnodontiforms through time reveals a pattern that matches that seen in their body shape changes (compare Marramà et al., 2016a) between the same time periods (Figure 4). There was a significant change in morphological disparity of pycnodontiform lower jaws throughout all time bins (*p* < 0.05). The morphospace shrinks in the Early Cretaceous only for it to greatly expand in the Late Cretaceous. The large increase in morphospace area and morphological disparity can be attributed to the appearance of dorso‐ventrally compressed and elongated jaws characteristic of †Gladiopycnodontidae and †Coccodontidae (Figure 4). There is another large reduction in both morphospace and disparity after the K/Pg boundary with just †Pycnodontidae remaining. Jaw morphology of dapediiforms and ginglymodians remains conservative, in comparison, with no significant changes through time.

Body disparity results among the four neopterygian groups show that Acanthomorpha is the most disparate clade followed by †Pycnodontiformes, Ginglymodi and †Dapediiformes. As with the lower jaw analysis, coccodontids have the highest while †Pycnodontidae has the lowest body disparity within †Pycnodontiformes.

### 3.5 Diversity

**FIGURE 6:**
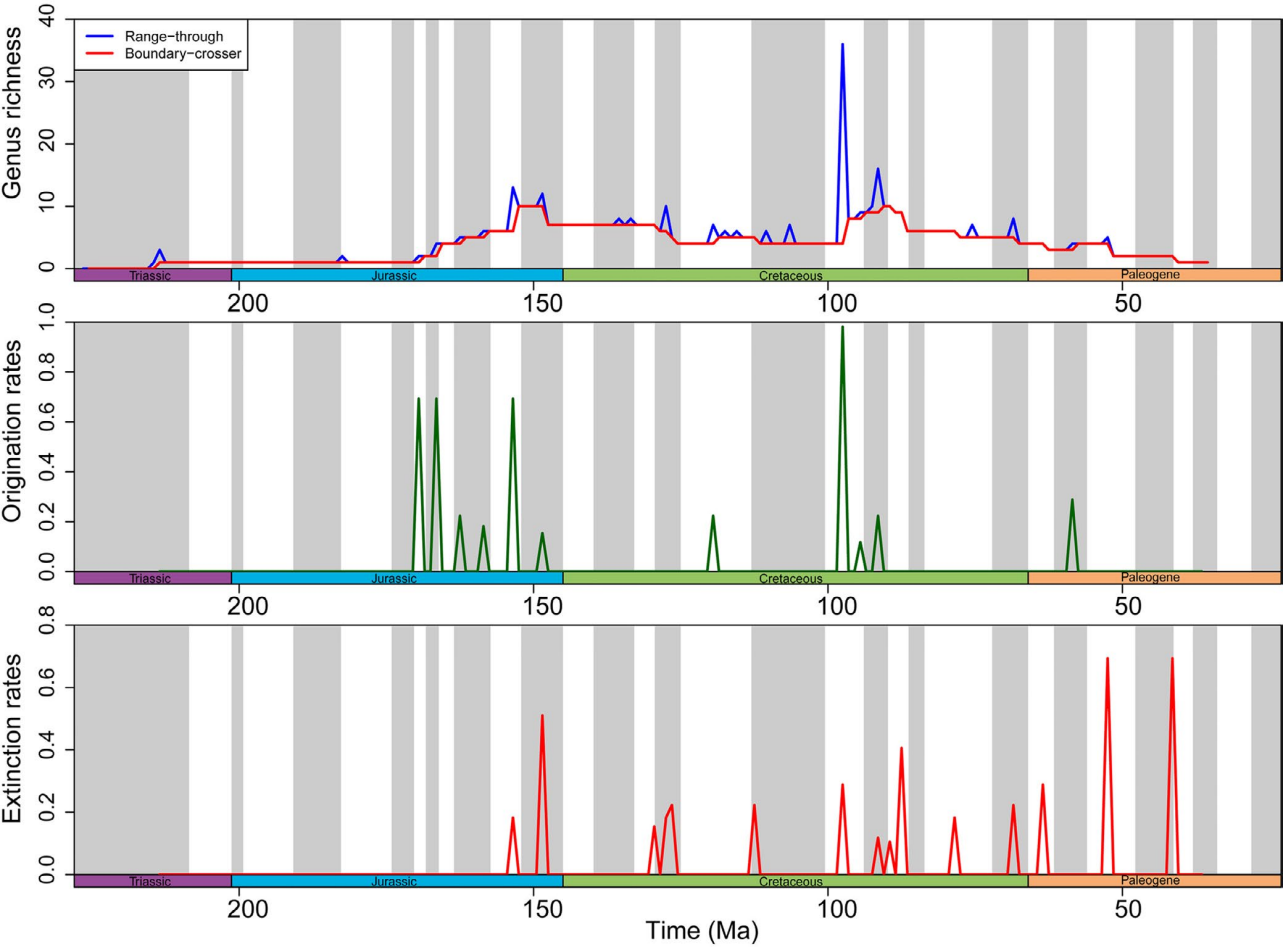
Diversity, origination and extinction rates of pycnodontiform fishes through time from the end of the Late Triassic (Norian) to the end of the Eocene (Priabonian)

Two peaks of high pycnodontiform diversity occur in the Late Jurassic (Kimmeridgian–Tithonian) and the Late Cretaceous (Cenomanian) (Figure 6). These peaks are present even with singletons removed indicating that these particular time periods were times of great diversification, as indicated by the origination rate peaks just before the diversity peaks. Curiously, pycnodontiforms were in decline before the K‐Pg extinction at least since the Coniacian and with the exception of a small spike in diversity in the remainder of the Cretaceous (late Campanian and Maastrichtian) and Late Palaeocene (Thanetian), they underwent a continuous decline until their final disappearance at the end of the Eocene. This is also evidenced by the series of spikes in extinction rates starting in the Late Cretaceous and continuing throughout the Palaeogene (Figure 6).

**FIGURE 7:**
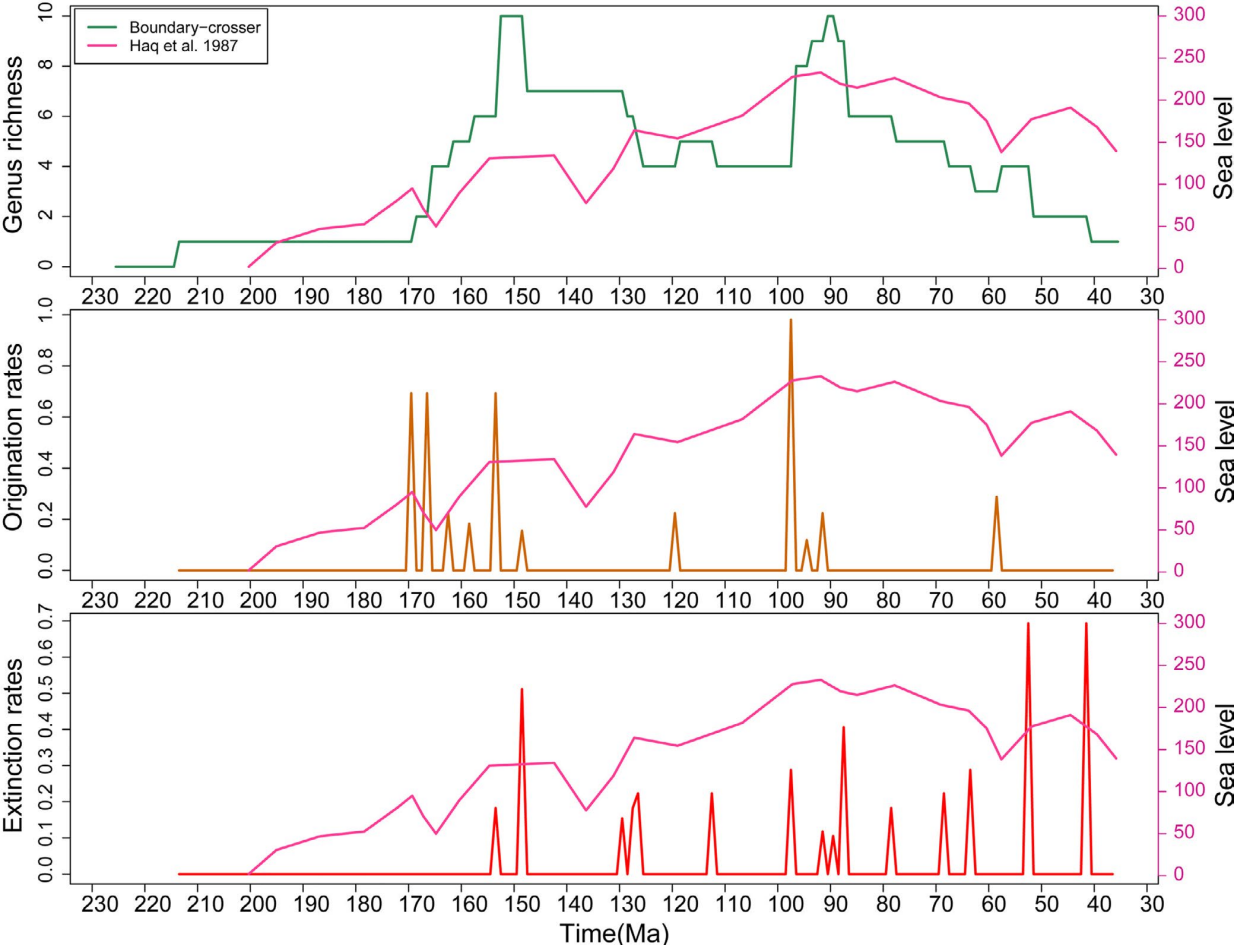
Diversity, origination and extinction rates of pycnodontiform fishes measured against sea level

The diversity and sea level curves do not follow a similar pattern (Figure 7). It is interesting to note that the Turonian rise in diversity lags the rise of sea level in the Cenomanian where sea levels were at their highest in the Mesozoic. The reduced diversity during the Albian is more indicative of collecting bias than a genuine decline in diversity. The other extensive diversity peak in the Late Jurassic was conversely, a time of comparatively low sea levels but large sea surface area.

**FIGURE 8:**
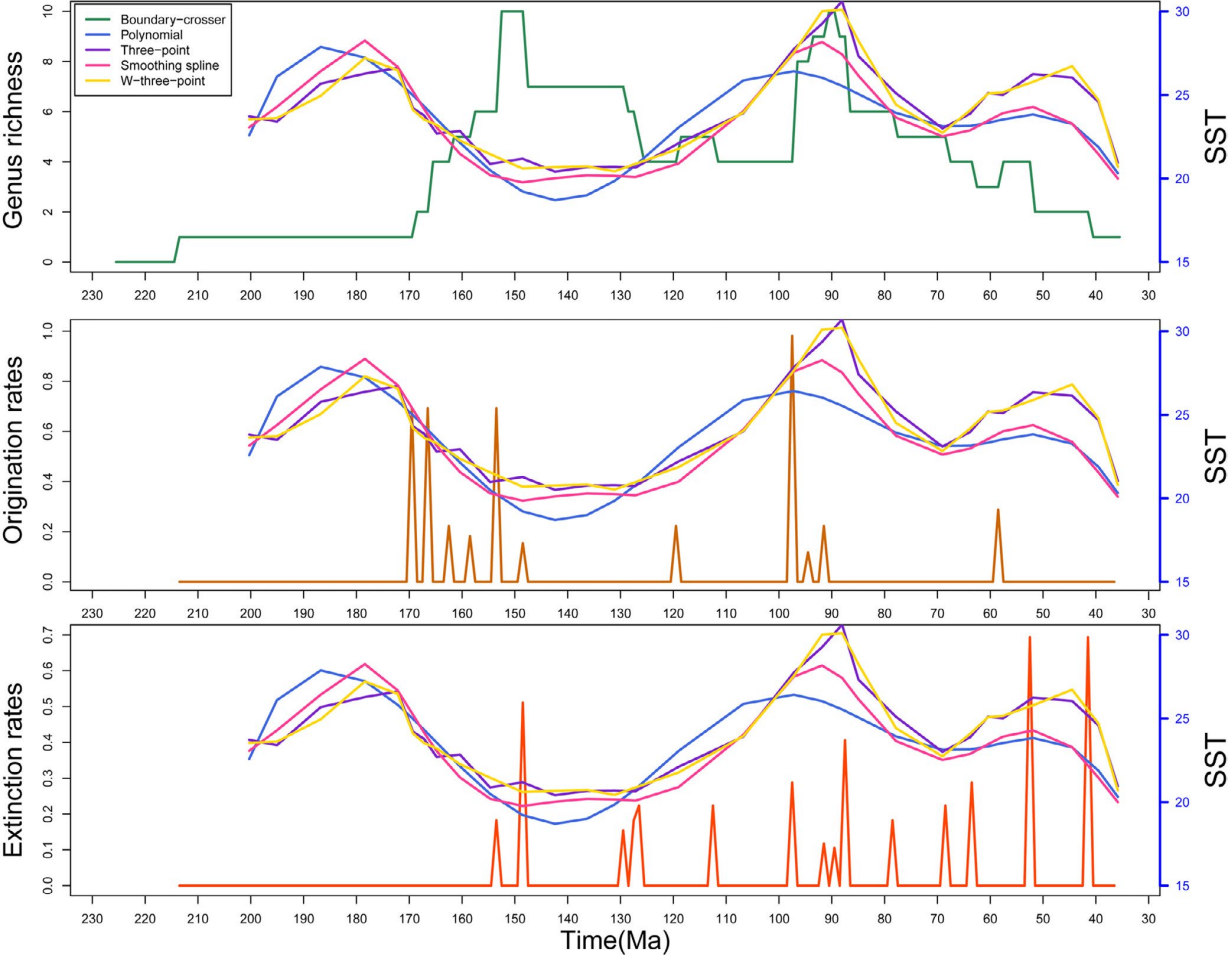
Diversity, origination and extinction rates of pycnodontiform fishes measured against sea surface temperature (SST). Weighted three‐point average is abbreviated to W‐three‐point. SST measurements range from the Hettangian to the Priabonian (Jouve et al., 2017)

Pycnodontiform diversity patterns also are not correlated with SST with a probable exception in the Cenomanian (Figure 8). Here, origination rates are positively correlated with an increase in ocean temperatures. The only other positive correlation between origination and SST is during the Thanetian when the Palaeocene‐Eocene Thermal Maximum (PETM) begins and SST steadily increases again. Like with sea level, high pycnodont diversity in the Late Jurassic is during a time of declining SST. The observed spike in extinction rates at PETM and toward the end of the Eocene when global cooling was occurring suggests that pycnodontiforms were heading toward extinction regardless of climatic changes as expressed by SST.

**FIGURE 9:**
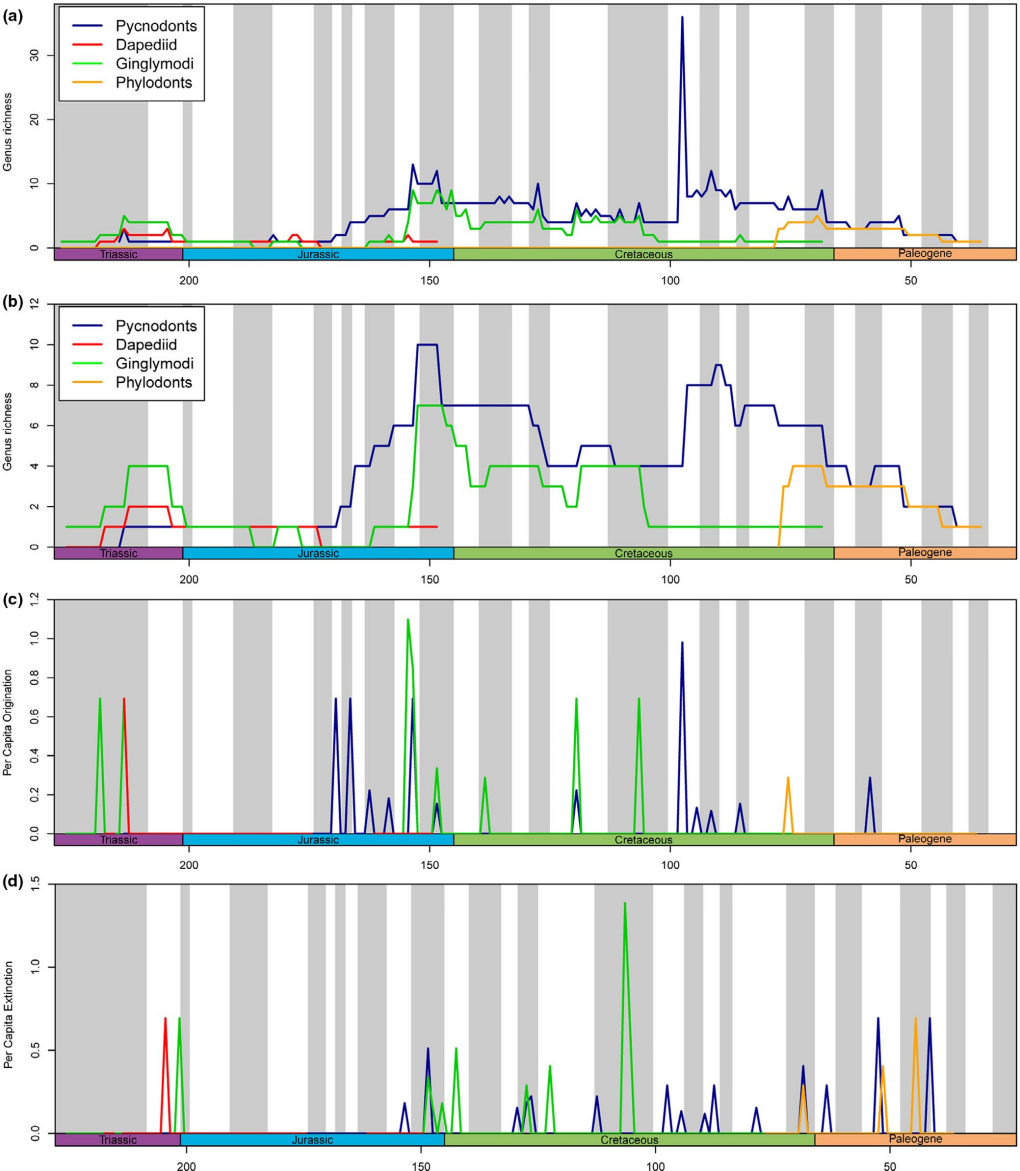
Diversity, origination and extinction rates of pycnodontiforms, ginglymodians, dapediiforms and phyllodontids through time. (a) All taxa including those restricted to a single time bin. (b) Only taxa that cross boundaries of time bins included. (c) Origination rates. (d) Extinction rates

Investigating diversity patterns of other Mesozoic fish clades reveals several findings (Figure 9). All groups experience low diversities for most of the Early to Middle Jurassic and both †Dapediiformes and particularly, Ginglymodi have rather high extinction peaks close to the Triassic‐Jurassic boundary compared to pycnodontiforms. †Dapediiformes only had one positive origination peak in the Late Triassic and an extinction peak closer to the Triassic‐Jurassic boundary with a smaller one in the late Norian (211.5 Mya). With the exception of the Toarcian where †*Tetragonolepis* was also present, only one dapediiform genus, †*Dapedium*, occurred throughout the Early to Middle Jurassic. However, despite the low generic diversity, †*Dapedium* is a speciose genus and its generalist nature could have enabled it to survive the tumultuous period of the Early Jurassic where suitable benthic habitat was rare (Kiessling et al., 1999, Figure 2) and anoxic events (Müller et al., 2017) occurred.

Ginglymodians were the most diverse of the three clades in the Late Triassic with them experiencing two remarkable origination events before the Triassic‐Jurassic boundary (Figure 9). Similar to †Pycnodontiformes, ginglymodians underwent a diversity burst in the Late Jurassic during the Kimmeridgian. The diversity curve of pycnodontiforms displays a series of high origination rates before the Late Jurassic starting in the Middle Jurassic (Bajocian). Ginglymodians, conversely, had a larger origination peak during the Kimmeridgian. Smaller overlapping origination peaks, but also an extinction peak for both †Pycnodontiformes and Ginglymodi occur in the Tithonian. Like pycnodontiforms during the Middle to Late Jurassic, ginglymodians experienced a series of positive origination rates during the Early Cretaceous. This coincides with ginglymodians becoming predominantly adapted to freshwaters providing new opportunities for diversification. Their final major origination event occurred in the Campanian. During the Albian, ginglymodians experience their largest extinction event yet, which resulted in the lepisosteids being the only remaining ginglymodians. A similar extinction burst for Ginglymodi is present in the Turonian.

Finally, †Phyllodontidae experienced their first major diversification event due to elevated origination rates close to their first occurrence in the fossil record in the Campanian (Figure 9). Two of the extinction peaks of their diversity curve overlap with those of pycnodontiforms, after the K/Pg extinction event in the Danian and the other in the Ypresian. The overall diversity patterns of †Pycnodontiformes thus appear to be independent of the patterns of the other Mesozoic clades suggesting that the rise or decline of other fish clades exerted no competitive pressure or release on pycnodontiforms.

## 4 DISCUSSION

### 4.1 Body and lower jaw disparity and its ecomorphological implications

The large morphological disparity and morphospace area displayed by pycnodontiforms support the hypothesis that this group occupied a wider range of ecological niches than that of other deep‐bodied shell crushers as already suggested by Poyato‐Ariza (2005a). Generally, Mesozoic nonteleostean groups are clearly separated into different parts of the body morphospace. Pycnodontiforms are predominantly concentrated into the upper left quadrant where deep‐bodied forms with comparatively small but elongate median fins reside. A few representatives of more derived families (†Coccodontidae, †Gladiopycnodontidae) occupy the upper right quadrant because their bodies are comparatively more elongated but the median fins are a constant feature. This suggests that pycnodontiforms are predominantly maneuvering specialists with the more elongate forms able to occupy more open spaces over the sea floor.

The morphological separation of †Pycnodontiformes from the other two nonteleostean clades in the lower jaw morphospace, Ginglymodi and †Dapediiformes, leads to the interpretation that the jaws were capable of performing different feeding actions from one another. The high coronoid process of many pycnodontiforms ensures that the adductor mandibulae muscles attached in a more vertical orientation, which increases the biting force (Kriwet, 2001a). Another factor that increased the biting force is the decreased length of the jaws (Gosline, 1965), which characterizes many of the pycnodontids on the positive values of RW2 axis such as †*Akromystax tilmachiton* and †*Proscinetes elegans*. The more vertical suspensorium also ensured that the adductor mandibulae muscles covered a smaller distance to attach to the jaw allowing more refined and efficient jaw movements.

Kriwet (2001a) discussed the possibility of antero‐posterior movements in the mandible of pycnodontiforms, which could not only enhance the nipping action by the dentalosplenial but also allows for a shearing movement in the mandible. Such jaw movements could enable pycnodontiforms to move into previously unexplored niches for actinopterygians such as flesh eating (Kölbl‐Ebert et al., 2018; Vullo et al., 2017). Additionally, many prearticular teeth had visible wear facets indicating that lateral directed shearing of the jaws was also possible (Baines, 2010). Further evidence that pycnodontiforms were capable of this lateral jaw movement is the rugose surface of the antero‐posterior elongated symphysis of the prearticulars that indicates the presence of possible connective tissue enabling such movements (Gosline, 1965; Kriwet, 2001a). Oral mastication in vertebrates is well known in mammals but similar feeding mechanisms seemingly have arisen numerous times in vertebrate evolution as in hadrosaur dinosaurs (Erickson et al., 2012) and stingrays (Kolmann et al., 2016). The elongated and high coronoid process is also a character commonly associated with mastication in tetrapods, for example, in ungulates (Hoshi, 1971) and ceratopsian dinosaurs (Ostrom, 1966). Tooth arrangement on the vomers and prearticulars along with possible masticatory jaw movements gave rise to the interpretation of pycnodontiform jaws as a grinding mill (Kriwet, 2001a) where prey was processed with increased efficiency compared to ginglymodians and dapediiforms. Conversely, the position of Ginglymodi and †Dapediiformes on the negative RW1 axis could be related to the fact that their adductor mandibulae muscles cover a greater portion of the head and were arranged more oblique similar to the condition in most other actinopterygians and would thus not have processed prey to the same degree or efficiency as pycnodontiforms, indicating that certain prey items, such as corals (see Applegate, 1992; Maisey, 1996), may have been inaccessible to these groups.

Further separation between the groups is expressed in body shape (Figure 2). Dapediiforms are also located within the “deep‐bodied and small median fin” morphospace (Figure 2) but their bodies are as a rule not as deep as in pycnodontiforms and they probably occupied more open habitats as they are less suited to maneuver around structured environments. Ginglymodians are on the right side of the morphospace (Figure 2) because they are more streamlined than the other two groups but two families, †Lepidotidae and †Macrosemiidae occupy different quadrants. The lepidotids in the upper right quadrant are generally bulky fishes with small median fins concentrated near the caudal fin while macrosemiids in the lower right quadrant are more streamlined with elongated dorsal and considerably smaller anal fins. Lepidotids would have been large and sluggish cruisers over sea bottoms and were most likely able to perform fine movements as they hover over attached prey and remove it or ambush prey. The elongated dorsal fin in some macrosemiids is surrounded by a scale free area (Ebert et al., 2016), which would have enabled them to undulate the dorsal fin resulting in more precise swimming and could even potentially have supported backwards as well as forwards swimming, as seen in some extant ray‐finned fishes such as *Amia* and *Gymnarchus* (Jagnandan & Sanford, 2013). Interestingly, the macrosemiids, which possess this scale free area such as *Macrosemius*, *Legnonotus*, *Palaeomacrosemius* and *Macrosemiocotzus* are located further along the positive RW2 axis where elongated dorsal fins are more predominant. This fin arrangement indicates that these fishes were slow swimmers but were adept at maneuvering around reef structures (Bartram, 1977), which would have been inaccessible to larger lepidotids. However, macrosemiids such as †*Legnonotus* could be capable of exploiting more open waters than either *Amia* or †Lepidotidae as suggested by their forked caudal fins.

### 4.2 Late Triassic

Our results also reveal how niche partitioning changes between and within the nonteleostean groups occurred through time and space. The taxa sampled here are from the Late Triassic (Norian) Zorzino Limestone of Lombardy, Italy (Lombardo & Tintori, 2005; Tintori, 1998) and are the first fossil occurrences for pycnodontiforms and dapediiforms with ginglymodians also present in sufficient numbers. †Brembodontidae occupies a quite different portion of lower jaw morphospace compared to derived pycnodontids, which appeared later in the fossil record (Figure 4). Morphological disparity of pycnodontiform lower jaws in the Late Triassic is higher than in the Jurassic. This is due to the occupation of two different quadrants in the lower jaw morphospace by †*Brembodus* and †*Gibbodon*. †*Brembodus* has a low, broad coronoid process that is shifted forward in position compared to later pycnodontiforms.

However, there is more than jaw shape to consider when interpreting the ecology of any of these fishes, especially the tooth type. The lower jaws of †*Brembodus* have a dentalosplenial with four unicuspid, elongate chisel‐like teeth and the prearticulars bear hemispherical to oval shaped molariform teeth (Poyato‐Ariza & Wenz, 2002; Tintori, 1981). This dentition suggests that †*Brembodus* was feeding on armored prey items such as molluscs, crustaceans and echinoderms. Another typical Late Triassic pycnodontiform is †*Gibbodon*, which already had the typical pycnodontiform jaw shape, displaying a dentition for a different diet. The dentalosplenial teeth are elongate and bicuspid (Kriwet, 2005), which is more ideal for scraping algae off rocks (Gibson, 2015). †*Gibbodon* also has peculiar papilliform vomerine teeth (J.J. Cawley, pers. obser.) that are tightly packed together in some ways resembling the pharyngeal tooth pattern of modern herbivorous cichlids (see Burress, 2016, Figure 1g). The use of oral dentition for prey capture and pharyngeal jaws for prey processing in these extant teleosts is similar to how †*Gibbodon* may have dealt with prey using its unique set of oral teeth. The combination of premaxillae and dentaries working together to scrape algae and the vomerine and prearticular being used as a mill to grind plant material would have been very effective for processing and assimilating such difficult prey items. Thus, we assume that herbivory might have been more common among pycnodontiforms than previously assumed (Baines, 2010; Darras, 2012). A recently described †*Pycnodus* premaxilla from the Moroccan Palaeogene also possesses multicuspid grasping teeth, which were shared by two other pycnodontiform taxa that occurred close to the K/Pg boundary (Vullo et al., 2019). Similar teeth are also found in †*Thiollierepycnodus*, †”*Eomesodon*” *hoeferi* and †*Nursallia tethysensis* (Capasso et al., 2009; Ebert, 2020).

Worthy of note is that dapediiforms, occupying the lower left quadrant of the jaw morphospace, are further to the positive end of the RW1 axis than ginglymodians, because their jaws are more dorso‐ventrally compressed indicating that they were not used to generate high bite forces. †*Sargodon tomicus* is the exception as it has a higher coronoid process. This taxon is the largest dapediiform in the Late Triassic, growing up to 1 m and has robust jaws with anterior chisel‐like teeth on the premaxillae and hemispherical and oval shaped grinding teeth on the prearticulars (Tintori, 1983). The combined size and shape of the jaw and teeth would make †*Sargodon* one of the most powerful shell crushers in the Late Triassic ecosystems that focused on larger prey than †*Brembodus*. The other two dapediiform species †*Dandya ovalis* and †*Dapedium noricum* possessed anterior slender pointed teeth along with densely packed, hemispherical teeth on the coronoids used for grinding on the prearticular.

In †*Dapedium* species from the Early Jurassic, the continuous battery of small rounded prearticular teeth are far too small to efficiently crush or grind shelled prey but would be useful in processing smaller, soft prey while the pointed marginal teeth would be used to bite, grasp and manipulate its food (Smithwick, 2015). Since specimens of †*Dandya ovalis* (Figure 1l) are of the same size range as pycnodontiforms, it seems likely that teeth of †*Brembodus* indicate a higher specialization on tougher prey items while †*Dandya* could have been able to feed on a wider variety of prey due to its deep jaws and pointed teeth. †*Dapedium noricum*, with its shallower jaws would most likely have concentrated on smaller soft prey items (Lombardo & Tintori, 2005). Dapediiforms differ little from pycnodontiforms in many respects of body shape but are generally more elongate. †*Sargodon*, however, is intermediate in body depth between pycnodontiforms and other dapediiforms suggesting that it probably was more specialized for maneuvering in structured habitats (Tintori, 1998).

Late Triassic ginglymodians are more elongated than either pycnodontiforms or dapediiforms in body shape but all three ginglymodian taxa occupy a continuum from deep‐bodied †*Semiolepis* with its median fins located near the caudal fin on one end, and the elongate macrosemiid †*Legnonotus* with elongated median and forked caudal fins. When considering the lower jaw among this group, however, an unusual pattern appears. †*Legnonotus krambergeri* has a high coronoid process but its stout, pointed teeth appear more suited for grasping pelagic shrimp than crushing shells (Lombardo & Tintori, 2005; Tintori, 1998). The coronoid process of †*Legnonotus* therefore indicates that it was durophagous but most likely would have focused on relatively small and more soft‐shelled prey items. Taking the results of the body and jaw morphospaces together, one can see that †*Legnonotus* was darting in among structured habitats such as reefs feeding on small crustaceans.

†*Paralepidotus ornatus* conversely, had powerful crushing dentitions and these fish became progressively more durophagous during life as specimens over 25 cm have far stouter, hemispherical teeth than juveniles (Tintori, 1996). †*Paralepidotus* was also intermediate in size between †*Brembodus* and †*Sargodon*, growing to a maximum of 50 cm. In the fish‐bearing layers at Zorzino (Italy), ejecta consisting of crushed shells (Jadoul, 1985) suggest that this ecosystem was very productive in terms of molluscs and ensured a diverse array of durophagous fishes as described above. However, the lower jaw depth of †*Paralepidotus* is intermediate between dapediiforms (excluding †*Dapedium noricum*) and †*Brembodus*. The lower jaw of †*Semiolepis* is the most dorso‐ventrally compressed of all Triassic ginglymodians and its pointed marginal teeth with rounded coronoid teeth suggest that it was the least specialized for durophagous feeding. †*Semiolepis* and †*Paralepidotus* (Lombardo & Tintori, 2008; Tintori, 1996) were most likely slow cruisers just above the sea floor feeding on benthic prey with †*Paralepidotus* tackling tougher prey than †*Semiolepis*. These results seem to suggest that teeth suitable for crushing appeared before the jaw and suspensorium changed in shape to be more suited for more forceful bites.

### 4.3 Jurassic

Since pycnodontiforms are extremely rare in the Early to Middle Jurassic, the ginglymodian †*Lepidotes* and †Dapediiformes are the only taxa that can be investigated in terms of morphology and both taxa are significantly separated from each other. †*Tetragonolepis* is the deepest‐bodied of the dapediiforms with a shorter caudal fin. A general shift toward more elongate bodies is observed in dapediiforms from the Late Triassic to the Jurassic indicating further specialization toward open waters with †*Dapedium caelatum* being the most streamlined of the group. In the morphospace, †*D. caelatum* is further along the RW1 axis indicating a reduction in body depth and thus adaptation to more open water habitats. Another indication of this trend is a shift in pectoral fin position from below the interopercle in earlier forms to above the interopercle in later forms (Maxwell & López‐Arbarello, 2018). High pectoral fins indicate pelvic fin reduction and are suggestive of steady swimming supporting, that is, pelagic lifestyles, while low pectoral fins along with large pelvic fins seems to indicate adaptations for a benthic life (Breder, 1926). Another difference between Jurassic dapediiforms and pycnodontiforms is that dapediiforms were found in mudstone deposits (Lord & Davis, 2010), which contain thin layers of black shale. These indicate that anoxic conditions were characteristic of the sea bottom (Hallam, 1964) and would have been hostile environments for typical benthic invertebrates to colonize. This suggests that dapediiforms had to exploit waters in the more productive upper layers and its generalized jaws would have enabled them to be successful within this environment. The rarity of pycnodontiforms in the Early Jurassic (Kriwet, 2001b; Stumpf et al., 2017) could be explained by these anoxic events preventing the formation of reefs and hardgrounds for which the pycnodontiforms were predominantly specialized. The more generalized †*Dapedium*, however, was able to thrive in such extreme environments (Smithwick, 2015). In †*Dapedium* species from the Early Jurassic, the continuous battery of small rounded prearticular teeth are far too small to efficiently crush or grind shelled prey but would be useful in processing smaller, soft prey while the pointed marginal teeth would be used to bite, grasp and manipulate its food (Smithwick, 2015). Although its jaws were well suited for durophagy as the quantitative functional analysis on its jaws shows (Smithwick, 2015), †*Dapedium* was also a highly generalist feeder, as indicated by one specimen found with the shell of the ammonite †*Lytoceras* (Thies & Hauff, 2011) and another with the small teleost †*Dorsetichthys* impaled on its marginal teeth (Smithwick, 2015). This opportunism would have presumably contributed to †*Dapedium* surviving the end‐Triassic extinction event.

Within ginglymodians, lepidotids are generally bulky fishes with small median fins located near the caudal fin. This arrangement of fins on the posterior trunk is similar to that seen in acceleratory fishes such as pikes and gars (Webb, 1984), which might have enabled more elongated forms such as †*Lepidotes* to quickly overtake swimming crustacean prey. This interpretation is also supported by the presence of marginal styliform teeth in †*Lepidotes* that would support catching evasive prey before crushing it with the palatal dentition and stomach contents of shrimp cuticles (Thies et al., 2019). The coronoid processes of †*Lepidotes*, which are lower than in †*Scheenstia* indicate a less developed biting force suggesting a preference for more evasive and moderately armored prey.

The Late Jurassic was the time when pycndontiforms started to become much more common in the fossil record. This certainly is also related to the presence of conservation Lagerstätten, which enabled the preservation of articulated specimens. Even if these represent singleton occurrences they also bear biological signals, as evidenced by the remarkably rich and diverse fossil record of pycnodontiforms (e.g., Ebert, 2013, 2016, 2020; Kölbl‐Ebert et al., 2018; Kriwet, 2001b; Poyato‐Ariza & Wenz, 2002). The pycnodontiform taxa examined herein are predominantly from the Plattenkalk deposits of the Late Jurassic Solnhofen Archipelago, which can provide phenotypic evidence both of the lower jaw and the whole body physiognomy. Ginglymodians are represented by the large lepidotid †*Scheenstia* and macrosemiids in the Late Jurassic. †*Scheenstia maximus* has the most anteriorly placed and highest coronoid process of all fishes in this particular ichthyofauna. †*Scheenstia maximus* is one of the largest ginglymodians in the Late Jurassic, growing to a standard length of over 1.5 m (López‐Arbarello, 2012) and maximum length just over 2 m long (Jain, 1984). This size, along with its smooth, rounded molariform teeth makes it a truly formidable shell crusher. †*Scheenstia*, conversely to †*Lepidotes* with its smaller median fins and large, rounded teeth would enable more precise control as it hovered over the seafloor removing attached shelled prey from the seafloor to crush. †Macrosemiidae such as †*Propterus elongatus*, †*Macrosemius rostratus* and †*Palaeomacrosemius thiollieri* seem to be relatively more adapted for durophagy than †*Propterus microstomus* as their coronoid processes are taller (Figure 1m).

Jurassic pycnodontiforms were more diverse in their lower jaw morphospace than in their body morphospace but there are subtle differences in body shape that indicate niche partitioning. Gyrodontids are positioned between dapediiforms and pycnodontids in terms of body depth, which suggests that they were adapted to more open water habitats than pycnodontids but simultaneously were more maneuverable than dapediiforms. This implies that these fishes were patrolling the reef edges but could also travel out into open water in search of suitable habitats, which could explain their wide distribution (Kriwet & Schmitz, 2005). †*Gyrodus* differs from †*Scheenstia* in its lower coronoid process morphology and presence of styliform dentalosplenial and premaxillary teeth. This morphology makes †*Gyrodus* less specialized and probably made it more of a generalist preying on less armored invertebrates and most likely had a broader trophic niche than †*Scheenstia*. †*Arduafrons prominoris* has similar jaw shapes and styliform teeth to †*Gyrodus* but its lozenge‐shaped body made it more suitable to swim among the structures of reefs and would have avoided competition with †*Gyrodus* in this way. The discovery of echinoid spines preserved within †*Arduafrons* (NHMUK P8658; Nursall, 1999) and †*Gyrodus hexagonus* (VFKO‐X 11; Kriwet, 2001a) specimens shows that both pycnodonts preyed on echinoderms. Nursall (1999) argued, using as evidence the presence of spines in the gut and the prognathous snout with eyes set back a considerable distance from said snout that †*Arduafrons* might have had feeding habits similar to extant triggerfishes, which disarm such spiny prey by breaking off the spines before swallowing the prey item (Fricke, 1971). Members of †Pycnodontidae have the most highly developed coronoid processes and shortest jaws among pycnodontiforms so they were probably the most specialized for durophagy in the group.

†*Piranhamesodon pinnatomus* has a larger biting area than all other pycnodontiforms due to a large dentalosplenial bone armed with sharp teeth which are interpreted to be used for removing chunks of flesh/pieces of fins from their prey (Kölbl‐Ebert et al., 2018). This species shows the typical pycnodontiform phenotype (deep body, posteriorly placed median fins) with a morphospace occupation similar to that of serrasalmids (piranha and allies; Burns & Sidlauskas, 2019). The rounded caudal fin and small backward‐facing median fins of †*Piranhamesodon* also indicate that it was a slower swimmer than both typical pycnodontiforms and modern pirahnas that possess more forked caudal fins. Kölbl‐Ebert et al. (2018) pointed out that the damaged fins and fin bases of large fossil fishes from the same locality as †*Piranhamesodon* could be evidence of this pycnodontiform removing pieces of fins from unwary fishes. Being a slow but maneuverable fish it could have been an aggressive mimic (Peckham's mimicry; Peckham, 1889) where it could blend in with more harmless fishes and get close to its prey to attack.

### 4.4 Early Cretaceous

In the Early Cretaceous, pycnodontids still occupied the same morphospace quadrant with †*Iemanja* being the furthest outlier with a shallower body and an elongated skull, which hints at it being more adapted for feeding in crevices (Cawley & Kriwet, 2019; Poyato‐Ariza, 2005a). Jaw morphospace has shrunk considerably for both pycnodontiforms and ginglymodians in the Early Cretaceous with only †*Stemmatodus* being an outlier from the typical pycnodontiform morphospace with slightly forward facing coronoid processes. Among ginglymodians, †*Macrosemiocotzus* possesses pointed, stout teeth on its jaws with none adapted for crushing prey (González‐Rodríguez et al., 2004) but possesses a highly developed coronoid process. Preserved stomach content of this species contains “copepod appendages, algal structures, and many unidentified palynomorphs” (González‐Rodríguez et al., 2004). Such a mismatch between feeding morphology and prey in this specimen is a pertinent example of Liem's paradox (Liem, 1980).

### 4.5 Late Cretaceous

Much of the disparity of pycnodontiform body and jaw shapes can be attributed to the famous Late Cretaceous Plattenkalk of Lebanon, which provided an incredible abundance of pycnodontiforms (Cawley & Kriwet, 2019; Marramà et al., 2016a; Nursall & Capasso, 2004; Poyato‐Ariza & Wenz, 2005; Taverne & Capasso, 2013a,2013b, 2014a,2014b, 2015a,2015b). The morphospace for this group is by far the largest of all time bins analyzed here (Figure 5). The Late Cretaceous morphospace occupation of †Pycnodontidae expands significantly during this time with †*Haqelpycnodus* representing a deep bodied form with a smaller head and comparatively long median fins and †*Tergestinia* producing a more streamlined form with a caudal fin with a straight margin and a distinctive caudal peduncle. Lebanese †Pycnodontidae have even higher coronoid processes than in any other locality indicating higher bite forces and that some truly specialized durophagous forms have appeared by this time as evidenced by their high scores on the RW1 axis. Conversely, pycnodontiform families on the negative RW1 axis such as †Gladiopycnodontidae have more elongate and shallow jaws with a reduced coronoid process. This morphology is indicative of smaller bite forces, which indicates that the jaws had a faster rather than a forceful bite (Albertson & Kocher, 2001). This is quite unusual for pycnodontiforms and indicates that gladiopycnodontids might have been feeding on more evasive prey. Combined with the more elongate body of these fish it is reasonable to hypothesize that gladiopycnodontids occupied a different niche than typical durophagous pycnodontiforms.

The Lebanese Plattenkalk seemingly was a cradle of diversity for bony fishes in general (Hückel, 1970), where pycnodontiforms reached their highest morphological disparity (Marramà et al., 2016a). The considerable environmental heterogeneity included broad areas with rudist mounds and patch reefs (Hemleben & Swinburne, 1991), providing ample opportunities for niche partitioning among pycnodontiforms allowing them to diversify and it seems to be the case that some gladiopycnodontids might have become adapted to more open environments to avoid competition with other pycnodontiform groups (Marramà et al., 2016a). The dentition of gladiopycnodontids also is peculiar. The premaxillary and dentalosplenial teeth are incisiform, which is typical for pycnodontiforms but the vomer contains a patch of tiny, rounded molariform teeth while the prearticular has larger oval and hemispherical molariform teeth (Taverne & Capasso, 2013a). The vomerine teeth are very peculiar for pycnodontiforms and are more similar to prearticular teeth of dapediiforms. Such an arrangement is different enough that they certainly were not targeting similar prey as members of †Pycnodontidae, which supports their complete separation in the Haqel morphospace.

Body shape morphospace occupancy reveals that †Gladiopycnodontidae had a diverse range of morphologies. Elongate forms such as †*Gladiopycnodus* and †*Joinvillichthys* overlap with the lepidotid ginglymodians suggesting a possible demersal lifestyle where they probably would feed on small benthic and demersal invertebrates hidden within the sand or mud. †*Rostropycnodus* and †*Ducrotayichthys*, conversely, would have been capable of maneuvering in more structured habitats but would have likely fed on more soft‐bodied prey than other pycnodontiforms.

†*Gebrayelichthys* surprisingly has a quite narrow and high coronoid process combined with slenderer anterior jaw portions (Figure 3). Investigating the few preserved teeth on the vomer reveals them to be small and conical with a pointed apex (Nursall & Capasso, 2004). However Taverne and Capasso (2014c) identify the vomer as a misplaced maxilla and the pointed teeth to be actually specialized spines of the maxilla. The only actual teeth of gebrayelichthyids preserved are those on the dentary and entopterygoid which have incisiform and small, rounded shapes respectively (Taverne & Capasso, 2014c). Nursall and Capasso (2004) suggested that such spiny structures could enable these pycnodontiforms to either target large zooplanktonic or slow swimming invertebrates such as comb jellies, pteropods and other pelagic gastropods, and free‐swimming tunicates. The high coronoid process is suggestive of higher bite forces, demonstrating that †*Gebrayelichthys* could securely hold onto such soft, slippery prey when it had been caught.

Gebrayelichthyids occupy a portion of the body morphospace that no other fish family occupies: extreme deepening of the body with small median fins positioned near the caudal fin. Such morphology is suggestive of a mid‐water dweller, which could have relied on camouflage to remain undetected from predators but this form of locomotion is also seen in fishes that live among reefs (Bartol et al., 2003). Its median fins would oscillate enabling the fish to swim without body flexure, because its body was too rigid otherwise due to large and extensive dorsal and ventral ridge scales lining the dorsal and ventral contour as well as the very short vertebral column (see Blake, 1977). The caudal fin was probably exclusively employed during a burst start or to escape quickly (see Lindsey, 1978). The spines on the posterior region between the dorsal and caudal fin would have deterred predators from attacking the slow swimmer. Fishes clustered high on the RW2 axis appear to be taxa that rely on the ostraciiform swimming mode and the arrangement and position of fins in gebrayelichthyids suggest similar locomotory adaptations. To further support this, only tetraodontiform fishes occupy this section of the morphospace although only in the Palaeogene. Thus, gebrayelichthyids may be tentatively considered a nonteleost lineage that evolved convergently an ostraciiform mode of locomotion.

The most streamlined pycnodontiforms are the coccodontids (†*Coccodus*, †*Corusichthys*) with antero‐posteriorly elongate bodies with small median fins. †*Trewavasia* and †*Hensodon* are at the other extremes with deeper bodies typical of pycnodontiforms.

This pattern repeats in the lower jaw with typical coccodontids having backwards shifting coronoid processes on a dorso‐ventrally compressed jaw, while †*Hensodon* and †*Trewavasia* have forward facing coronoid processes with the former having a low coronoid process while the latter has a high one. Coccodontids seem to be adapted for a similar habitat as the more elongate gladiopycnodontids but could possibly feed on tougher prey due to possessing a comparatively higher coronoid process. Of particular interest in this time period is the minimal overlap between pycnodontiforms and acanthomorphs in body morphospace occupancy. While acanthomorphs have a wide variety of both fusiform and deep‐bodied forms compared to pycnodontiforms, they have larger median fins and forked caudal fins. The only exception to this is the putative tetraodontiform †*Plectocretacicus clarae*, which has a deep body with small median fins restricted to the posterior trunk typical of the order. This suggests that there was significant niche partitioning in regards to habitat occupation between pycnodontiforms and acanthomorphs, with pycnodontiforms probably occupying more structured environments and feeding on hard‐shelled prey items or even algae, while fusiform acanthomorphs were adapted to more open waters. Deep‐bodied acanthomorphs, however, were not durophagous since none of these teleosts developed crushing‐ or grinding‐type dentitions.

### 4.6 Palaeogene

In the Palaeogene, the morphospace for pycnodontiforms shrinks rather dramatically after the K/Pg boundary extinction event. †Pycnodontidae is the only family present and †*Pycnodus* had a more streamlined body but not to the extent that the Late Cretaceous †*Tergestinia* had. In contrast to the deep‐bodied juveniles, the streamlined adults of *Pycnodus* were probably cruising over the reef but rarely hiding within the reef structure itself (Cawley et al., 2018). Morphological disparity is higher when it comes to the lower jaw with †*Pycnodus* having a far higher coronoid process than †*Nursallia* indicating it as a durophagous specialist, which is supported by a specimen discovered with numerous bivalve shells in the region where the digestion track would have been (MNHN Bol 135; Kriwet, 2001a). When pycnodontiforms have been found with stomach content, the prey consumed is usually monospecific, be it bivalve, coral or echinoderm (Kriwet, 2001a).

As shown in previous analyses (Marramà et al., 2016b), the acanthomorph morphospace expands considerably in the Palaeogene covering all four quadrants and now completely overlaps with that of pycnodontiforms. Interestingly, this overlap with pycnodontiforms is characterized by only two acanthomorphs, †*Vomeropsis* and †*Massalongius*. While they are both deep‐bodied fishes (more similar to †*Pycnodus* than to †*Nursallia*), which most likely cruised above the reef, their jaws and dentition reveal that these fishes were unlikely competitors of pycnodonts; †*Massalongius* was a benthic precision feeder characterized by delicate teeth while the putative carangid †*Vomeropsis* had protrusible jaws used for sucking in evasive prey. So while acanthomorphs had moved onto the reef and lived alongside pycnodontiforms, it is evident that they were feeding on different prey and thus competition over such resources can be considered unlikely. Therefore, competition by Acanthomorpha might not have been the factor that drove pycnodontiforms into extinction. In addition, durophagous acanthomorphs with dentition similar to that of pycnodontiforms, including the truly durophagous members of the family Sparidae did not appear until the Oligocene (Santini et al., 2014) after pycnodonts went extinct.

### 4.7 Pycnodontiform success and environmental factors

The lack of any correlation of pycnodontiform diversity patterns with abiotic variables such as SST and sea levels (Figures 7, 8) was already detected by previous studies (e.g., see Cavin et al., 2007). We nevertheless re‐evaluated such relationships as the last studies were conducted before several major contributions to pycnodontiform taxonomic diversity were known such as †Gladiopycnodontidae (Taverne & Capasso, 2013a) and †Serrasalmimidae (Vullo et al., 2017) as well as the many recently discovered taxa of †Pycnodontidae (Ebert, 2016, 2020; Poyato‐Ariza, 2010; Taverne & Capasso, 2013b, 2018a, 2018b; Taverne et al., 2015; Taverne et al., 2019, 2020; Cawley & Kriwet, 2018, 2019). Some of the highest diversities of †Pycnodontiformes are present in the Late Jurassic, which was a time of low sea levels but expanded seas. Most diversity of Late Jurassic pycnodontiforms and ginglymodians arise from the Plattenkalk deposits of the Late Jurassic Solnhofen Archipelago. These deposits originated in a series of distinct marine basins separated from the open ocean by barrier reefs (Barthel, 1970), which would have been ideal conditions for high fish diversity as each basin would have had specific ecological conditions with its own specially adapted fauna. In the Cenomanian–Turonian, pycnodontiform diversity is positively correlated with both sea level and SST indicating that these factors in combination may have triggered an increase in origination rates and thus in taxonomic diversity. That both sea level and SST are at their highest during the Cenomanian seems to be the result of a rise of oceanic crust production and/or oceanic volcanism (Gale, 2000). The Western Tethys Ocean was the center of origin for pycnodontiforms since their first recorded appearance in the Late Triassic (Poyato‐Ariza & Martín‐Abad, 2013) and this pattern repeats in the Late Cretaceous when certain pycnodontiform lineages such as the gebrayelichthyids (Nursall & Capasso, 2004) and gladiopycnodontids (Taverne & Capasso, 2013a, 2014a, 2015b) first appear in the fossil record.

**FIGURE 10:**
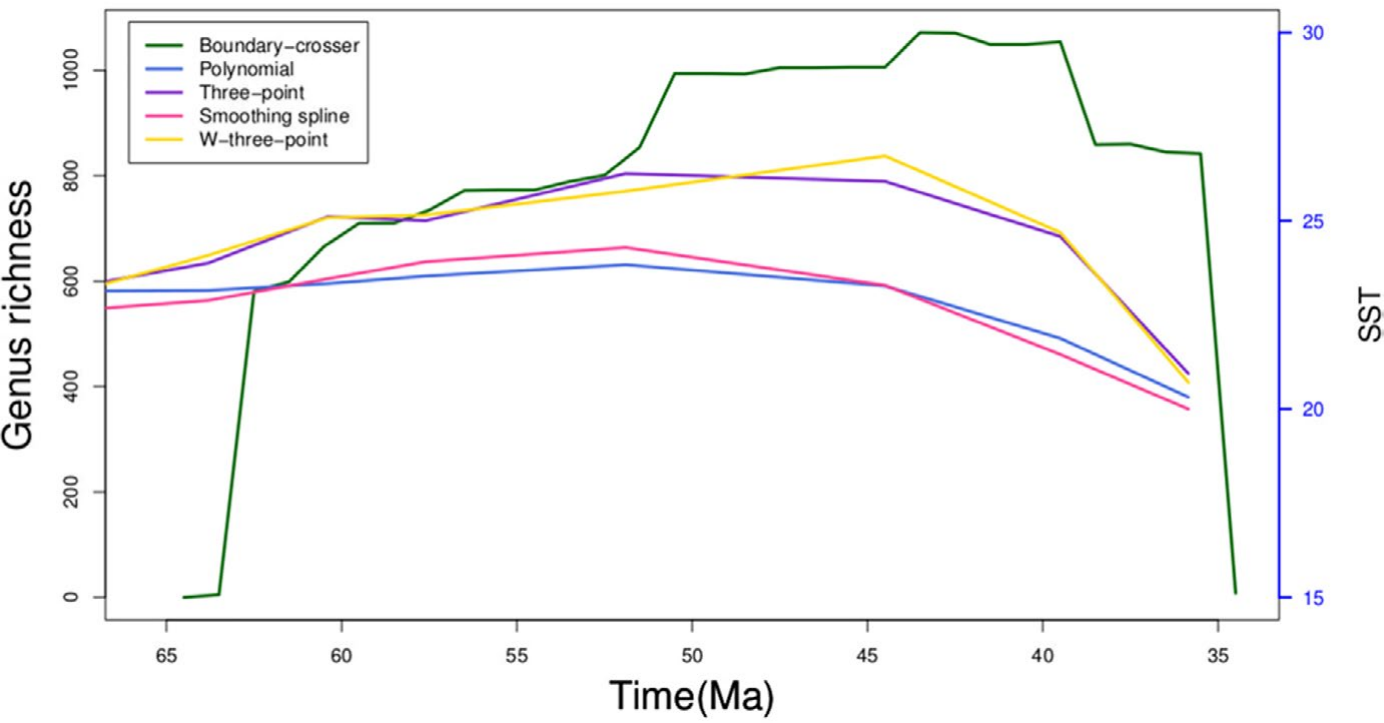
Diversity of shelled marine invertebrates (Echinodermata, Brachiopoda, Bryozoa and Mollusca excluding shell‐less cephalopods) from the Palaeogene measured against SST

Cavin et al. (2007) hypothesized that the rise in oceanic crust production contributed to the rise in this diversity as this could have led to an increase in area to be colonized by reefs. The number of reefs through time actually provides a more promising correlation with diversity patterns of pycnodontiforms. Reef numbers were lower during the Early Jurassic (Kiessling et al., 1999, Figure 2), which could explain the low pycnodont diversity in this time interval, while it was higher during the Late Jurassic, which also led to a significant rise in pycnodontiforms richness despite the decline in both SST and sea level (Figures 7, 8). The high genus richness in the Late Cretaceous (Cenomanian–Turonian) also correlates with a considerable expansion of reef areas (Kiessling et al., 1999, Figure 2). The reduction of reefs from the middle Campanian into the Palaeogene also marks a similar decline in pycnodontiform numbers.

The association of pycnodontiforms with a deep and short‐bodied morphospace along with the suggestion of diversity having a positive correlation with the number of reefs indicates an overall specialization for reef habitats and that the reduction of such habitats before and across the K/Pg extinction event continuing into the Palaeogene could have been a more likely culprit in their demise than either competition or other abiotic factors. Additionally, the decrease in pycnodontiform diversity during the Early Jurassic and the Palaeogene could also be affected by the two mass extinctions that occurred at the end of the Late Triassic and Late Cretaceous. Many reefs were wiped out during the Late Triassic extinction event so that the strong decline of reefs (84% ± 11%; Flügel & Kiessling, 2002) is referred to as the “Triassic‐Jurassic” or “Early Jurassic Reef Crisis” (Kiessling & Simpson, 2011). The total extinction of rudist bivalves, which were significant reef builders in the Late Cretaceous, at the K/Pg boundary may explain the continuous decline in pycnodontiform genus richness that is observed in our analysis.

But if Palaeogene reef numbers were far lower than during the Late Jurassic and most of the Cretaceous, then how is there such an explosion in diversity of acanthomorphs after the K/Pg extinction event? The acanthomorphs probably occupied a wide variety of biomes during the Cretaceous and many lineages survived the K/Pg extinction event to the degree that they replaced other neopterygian lineages that were dominant in certain environments up to the K/Pg boundary (Friedman, 2010). Although acanthomorphs, especially the highly diverse percomorphs, are certainly extremely abundant in reef ecosystems today as they were at least in the last 50 million years, their explosive radiation in the aftermath of the K‐Pg extinction was not primarily driven by the increased availability of reef habitats. The Palaeogene diversification of percomorphs resulted in the occupation of a vast spectrum of aquatic biotopes other than reef habitats (Friedman, 2010). While pycnodontiforms had representatives in brackish and freshwater environments (Cavin et al., 2020; Poyato‐Ariza, 2005b; Poyato‐Ariza et al., 1998), the vast majority found so far are in reef environments and were thus more susceptible to reef loss than acanthomorphs were. The few reefs that formed in the Palaeogene had more open niches available due to the reduction of pycnodontiforms which had an apparent stranglehold on these environments in the Late Cretaceous and thus, acanthomorphs could move in and undergo one of the most spectacular evolutionary radiations of vertebrates during the Cenozoic (Friedman, 2010).

The Eocene was a period of continuous pycnodontiform decline and the highest peaks in their extinction rates, one during the Ypresian and the other in the Priabonian, occurred at these times. The first extinction peak was during the PETM event, where ocean temperatures are estimated to have increased by a range of 4–8°C (Thomas et al., 2002) along with a rise in ocean acidification (Penman et al., 2014). Such conditions resulted in increased benthic extinctions (Thomas, 1998) and would have had an effect on the shelled invertebrates, which probably was the main food of pycnodontiforms. However, our diversity analysis of Palaeogene shelled invertebrates is not concomitant with climatic changes or pycnodontiform diversity patterns. It, therefore, is an unlikely factor in pycnodontiform extinction (Figure 10). From the Late Eocene going into the Oligocene, ice sheets were forming at the South Pole and the oceans were getting cooler (Liu et al., 2009). At this point in time, we assume that pycnodontiforms were a “dead clade walking” and after all these intense environmental changes died out in the Late Eocene. While acanthomorphs (particularly percomorphs) did not play a direct role in the extinction of pycnodontiforms, their ability to switch from one feeding mode to another (biting to ram and suction; Wainwright & Bellwood, 2002) enabled them to occupy a wider range of habitats, making them more versatile in this period of ocean changes and thus they were able to take advantage of these changes in a way that pycnodontiforms could not.

## 5 CONCLUSIONS

1. The majority of pycnodontiforms were specialized for maneuverability in reef‐like environments with only few forms adapted also to open water habitats (e.g., †Gyrodontidae) and had different jaw structures, which avoided any potential competition with dapediiforms and ginglymodians.
2. While both dapediiforms and ginglymodians overlap in jaw morphology their different body shapes indicate that they most likely occupied different niches or microhabitats thereby excluding any possible competition.
3. The differences between pycnodontiforms and other neopterygian lineages including durophagous forms could be related to improved jaw performance for feeding on tougher organisms. Altered feeding mechanisms such as mastication seemingly were improved in pycnodontiforms for prey processing, while the ontogenetic increase of the size of the fish allowed access to different types of prey and/or specialize on one or a few species of prey.
4. Pycnodontiform families also separate from each other in both the jaw and body morphospaces showing that they were most likely more diverse in their diets and habitat occupation than traditionally assumed. Gladiopycnodontids represent a significant expansion of pycnodontiform jaw morphospace, which is interpreted here as them occupying a more open, demersal habitat with new types of prey requiring different jaw morphologies and related soft structures such as muscles and ligaments. Hence the elongate, dorso‐ventral compact jaws most likely were used for picking small benthic prey off the substrate. Our analyses show that pycnodontiforms reduced competition with similar durophagous lineages by modifying the structures of the jaws for mastication of prey and further modification allowed pycnodontiforms to target different prey. While in larger taxonomic units, the difference in disparity is lower (†Pycnodontiformes, Ginglymodi) the differences are larger between families, sometimes even significantly (†Dapediidae, †Brembodontidae, “†Trewavasiidae”), which is indicative of further niche partitioning.
5. Comparing the diversity patterns of different Mesozoic fish groups also reveals that pycnodontiforms were not negatively or positively affected by diversity patterns in other clades, further indicating that competition between these groups was minimal or even absent. Competition with acanthomorphs in terms of body shape was minimal in the Late Cretaceous with pycnodontiforms restricted to more structured habitats, while acanthomorphs inhabited the biotopes between and outside such structures.
6. By the Palaeogene, acanthomorphs had significantly expanded their morphological disparity to the point that many representatives shared the same body shape with pycnodontiforms. However, competition with pycnodontiforms in terms of feeding ecology was highly unlikely, as teleosts do not show a genuine radiation of extreme durophagous forms until the Oligocene after the Eocene climatic optimum event and after pycnodontiforms went extinct. Consequently, we rule competition with acanthomorphs as the reason for pycnodontiform extinction out. Conversely, morphospace results show that pycnodontiforms may have kept acanthomorphs out of reef habitats and it was due to habitat loss in the Late Cretaceous that acanthomorphs experienced their rapid speciation during the Palaeogene. With pycnodontiform diversity already in decline, new niches were open for these more recent neopterygians to fill and we hypothesize that when acanthomorphs started to dominate reef fish communities, pycnodontiforms were effectively a “dead clade walking”, becoming a victim to background extinction rather than any significant environmental changes or absence of possible prey. Rather than pycnodontiforms being outcompeted by the more derived teleosts, it appears that teleostean fishes only took over reefs after pycnodontiforms were already beginning to decline and only developed durophagous forms after their final extinction.
7. The decline of reefs, particularly the extinction of rudist reefs, during the Late Cretaceous could be a promising avenue for future research in regards to abiotic and biotic drivers of pycnodontiform decline and extinction.

## CONFLICT OF INTEREST

None declared.

## AUTHOR CONTRIBUTIONS

**John J. Cawley:** Conceptualization (lead); data curation (lead); formal analysis (lead); funding acquisition (equal); investigation (lead); methodology (lead); visualization (supporting); writing – original draft (lead); writing – review and editing (lead). **Giuseppe Marramà:** Conceptualization (supporting); data curation (supporting); funding acquisition (equal); writing – review and editing (supporting). **Giorgio Carnevale:** Conceptualization (supporting); funding acquisition (equal); writing – review and editing (supporting). **Jaime A. Villafaña:** Formal analysis (supporting); methodology (supporting); visualization (lead). **Faviel A. López‐Romero:** Formal analysis (supporting); methodology (supporting); writing – review and editing (supporting). **Jürgen Kriwet:** Conceptualization (lead); funding acquisition (equal); supervision (lead); writing – review and editing (supporting).

## Data Availability

All data for this study is available in the Dryad Digital Repository: https://doi.org/10.5061/dryad.gtht76hk0.
